# Association of modifiable risk factors with obstructive sleep apnea: a Mendelian randomization study

**DOI:** 10.18632/aging.205288

**Published:** 2023-12-11

**Authors:** Ye Li, Yuyang Miao, Jin Tan, Qiang Zhang

**Affiliations:** 1 Department of Geriatrics, Tianjin Medical University General Hospital, Tianjin 300052, China; 2 Tianjin Geriatrics Institute, Tianjin 300052, China

**Keywords:** obstructive sleep apnea, modifiable risk factors, lifestyle behaviors, Mendelian randomization

## Abstract

Background: The risk factors involved in obstructive sleep apnea (OSA) have not been clearly identified yet. We attempted to systematically investigate genetically predicted modifiable risk factors and lifestyle behaviors associated with OSA.

Methods: The association between 34 risk factors and OSA was evaluated using the two-sample Mendelian randomization (MR). Genetic variants for risk factors were acquired from European-descent genome-wide studies. Data sources for OSA were extracted from FinnGen study with 16,761 cases and 201,194 controls. The primary analysis chosen was the inverse-variance weighted method.

Results: MR analyses provide evidence of genetically predicted poor overall health rating (odds ratio (OR), 2.82; 95% confidence interval (CI), 1.95–4.08), nap during day (OR, 2.01; 95% CI, 1.37–2.93), high body mass index (BMI) (OR, 1.14; 95% CI, 1.09–1.19), increased body fat mass (OR, 1.83; 95% CI, 1.83–2.05), elevated body water mass (OR, 1.50; 95% CI, 1.31–1.70) and hypertension (OR, 1.81; 95% CI, 1.34–2.45) were associated with higher OSA risk, while high education level (OR, 0.55; 95% CI, 0.40–0.75) correlated with reduced OSA risk. Suggestive evidence was obtained for smoking and waist-to-hip ratio (WHR) with higher OSA odds, and vigorous physical activity, and HDL cholesterol with lower OSA odds. After adjusting for BMI using multivariable MR analysis, the effects of smoking, WHR, vigorous physical activity, and HDL-cholesterol were fully attenuated.

Conclusions: This MR study indicates that overall health rating, nap during day, BMI, body fat mass, body water mass, hypertension, and education are causally associated with the risk of OSA, which means that these modifiable risk factors are key targets for OSA prevention.

## INTRODUCTION

Obstructive sleep apnea (OSA) is a globally prevalent disease that manifests as the repeated collapse of the upper airway during sleep. Among adults, approximately 14% of men and 5% of women have OSA and exhibit excessive sleepiness, which leads to decreased quality of life and a higher risk of vehicle accidents [1–3]. In addition, OSA may lead to an increased risk of developing hypertension, type 2 diabetes, atrial fibrillation, heart failure, stroke, Alzheimer’s disease and death [4–6]. Given that effective treatments for OSA are primarily lifestyle interventions, medical devices, and surgery, elucidation of risk factors for OSA is necessary [7]. This will contribute to reducing the medical and financial burden of early recognition of risk factors and associated cardiovascular disease comorbidities in patients with OSA.

Currently, there is a substantial body of evidence from observational studies and meta-analyses demonstrating the association between lifestyle factors and OSA, such as smoking, alcohol consumption, dietary habits, and exercise routines [8–15]. Also, lipid profile and metabolic syndrome-related factors (e.g., obesity, hypertension, and diabetes) are thought to be strongly associated with OSA risk in various studies [16–20]. Observational studies indicated that risk of OSA was also related to gender-related hormones [21], where progesterone may protect premenopausal women and higher androgen levels may raise OSA risk [22, 23]. However, investigating the causal association between these risk factors and OSA is a challenge due to the potential involvement of confounders and reverse causality in traditional observational studies. Therefore, the association has not yet reached a consistent conclusion. It is necessary to elucidate whether they have a cause-effect role in the pathogenesis of OSA or are simply a result of the shared risk factor profile. A clearer picture of which modifiable risk factors and OSA are causally linked could facilitate the determination of underlying goals for the prevention of OSA and ultimately cardiovascular disease.

Mendelian randomization (MR) design is a novel approach to causal inference using genetic variants of interest as instrumental variables [24], with independence from confounders and reverse causality [25]. The causal inference is further strengthened by the fact that genetic variants are randomly assigned during meiosis and remain unchanged across the lifespan. To date, limited MR studies have concentrated on the relationship between modifiable risk factors and lifestyle behaviors and OSA. The objective of this study was to use two-sample MR analysis to explore the causal association between 34 risk factors and OSA based on large population using summary-level statistics from genome-wide association studies (GWASs), and to further adjust for the potential effects of BMI or fat-free mass on some risk factors using multivariable MR analysis.

## METHODS

### Study design

**Figure 1:**
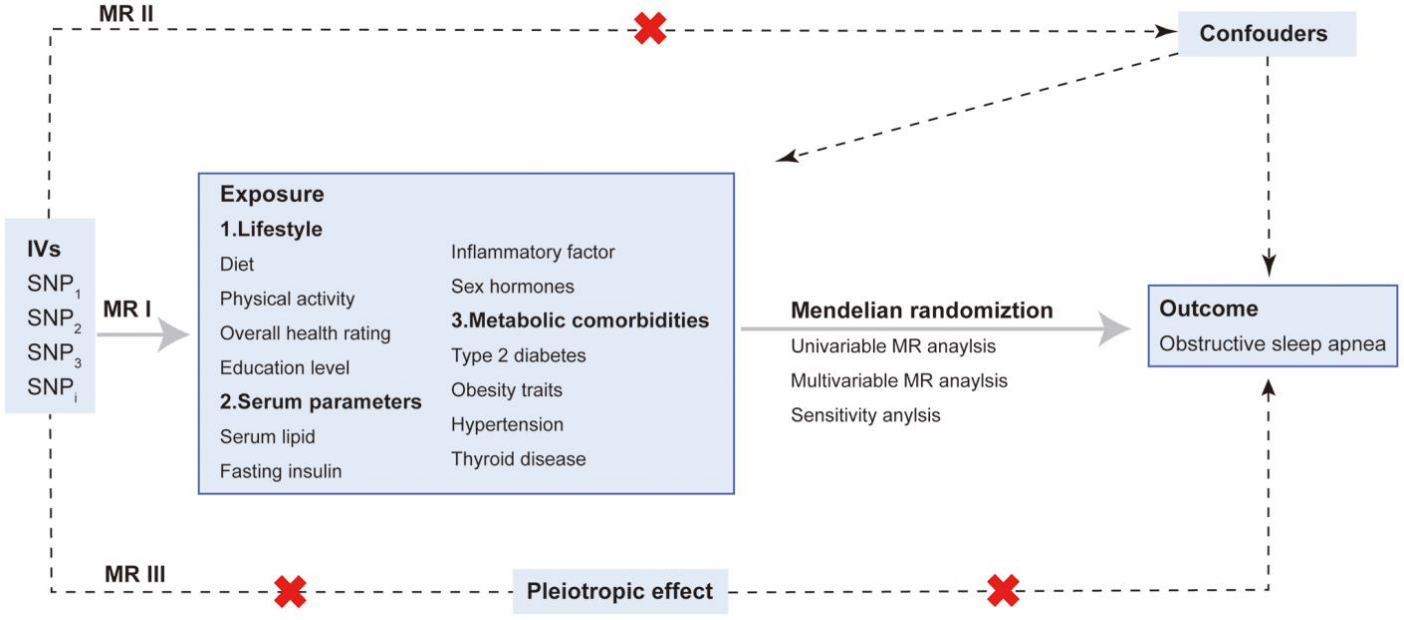
**Overview and assumptions of the Mendelian randomization study design.** The MR design was used to explore the causal association between four groups of risk factors and OSA, including lifestyle, serum parameters, metabolic comorbidities and sex hormones. The MR design satisfies three major assumptions: MR I, SNPs are strongly correlated with risk factors; MR II, SNPs are irrelevant to confounders; MR III, SNPs affect outcome merely via exposure. Abbreviations: IV: instrumental variable; SNP: single nucleotide polymorphisms.

The MR design used single-nucleotide polymorphisms (SNPs) as instrumental variables for risk factors. In order to make the causal effect to be nearly unbiased, it is crucial that the three major assumptions of MR are satisfied. The selection of SNPs followed the assumptions: (i) genetic instrumental variables are strongly correlated with risk factors; (ii) genetic instrumental variables are not associated with confounders of the exposure and outcome association; (iii) genetic instrumental variables are independent of the outcome, conditional on the exposure [26] (Figure 1). Altogether, 34 modifiable risk factors were included in present study and grouped into the following four groups: lifestyle, serum parameters, and metabolic comorbidities. The data used in this study were publicly available and no additional ethical support was required.

### Data sources and instrumental variables

Instrumental variables for modifiable risk factors were obtained in the largest GWAS performed in European ancestry. An overview of the data sources for the instrumental variables is available in Table 1. We selected SNPs that were associated with modifiable risk factors at genome-wide significance thresholds (*P* < 5 × 10^−8^) as instruments. The included SNPs were located in different gene regions and had less possibility of linkage disequilibrium (*r*^2^ < 0.001) with a long physical distance (≥10,000 kb). The risk factors contained in the four groups are as follows: lifestyle including diet, exercise, sleep, education and overall health rating; serum factors including lipids, C-reactive protein; testosterone and oestradiol; and metabolic complications including diabetes, obesity-related traits, thyroid disease, hypertension, body fat and water mass. Since few SNPs were associated with relative fat intake at a threshold of *P* < 5 × 10^−8^, we set the instrumental variable as *P* < 1 × 10^−5^. The GWASs selected for BMI have the advantage of no sample overlap with the other risk factor datasets selected, which is important for avoiding SNP-covariance estimation problems in multivariable MR analyses [27]. We utilize the F-statistic to assess the presence of weak variable bias of the instrumental variables, following equation: *F = R^2^/(1-R^2^) × (N-k-1)/k* [28] (Table 1). An F-statistic of greater than 10 implies that results based on reliable instrumental variables are less likely to be affected by weak instrumental bias [28]. The *R*^2^ is the proportion of risk factor variability explained by genetic instruments, *N* is the sample size, and *k* is the number of instrumental variables. R^2^ for each instrument variant: R^2^ = 2 × EAF × (1−EAF) × β^2^, where EAF is the effect allele frequency [29]. In multivariable MR, we use the conditional F-statistic to estimate the instrumental variables strength of exposures in the model [27]. We performed a statistical power analysis utilizing the online web tool [30]. In simple terms, it calculates the statistical power by taking into account the sample size of GWAS, the ratio of cases to controls, and the variance explained by the instruments of the exposure. The leave-one-SNP-out analysis was conducted to assess the influence of individual variants on the observed associations. Lastly, MR analysis was performed for each of the 34 eligible modifiable risk factors. The phenotypic variance interpreted by instrumental variables ranged from 0.16% for relative carbohydrate intake to 11.26% for high-density lipoprotein cholesterol (HDL-c) (Table 1).

**Table 1:** Characteristics of the GWAS summary data.

**Risk factor**	**SNPs**	**Sample**	**Population**	**F-statistic**	**Consortium**	**PMID**
**Diet**						
Alcoholic drinks per week	33	335,394	European	50.68	GSCAN	30643251
Smoking initiation	84	607,291	European	97.82	GSCAN	30643251
Cigarettes per Day	22	337,334	European	134.55	GSCAN	30643251
Coffee intake	38	428,860	European	73.15	UK Biobank	NA
Relative carbohydrate intake	10	268,922	European	40.30	SSGAC	30643258
Relative fat intake	45	268,922	European	28.00	SSGAC	30643258
Relative protein intake	6	268,922	European	55.11	SSGAC	30643258
**Physical activity**						
Number of days/weeks of vigorous physical activity 10+ minutes	11	440,512	European	39.68	UK Biobank	NA
Number of days/weeks of moderate physical activity 10+ minutes	16	440,266	European	35.99	UK Biobank	NA
Sedentary	4	103,712	European	33.48	UK Biobank	30531941
Nap during day	84	462,400	European	45.79	UK Biobank	NA
**Physical condition**						
Overall health rating	103	460,844	European	41.09	UK Biobank	NA
**Education**						
Education level	38	307,897	European	37.49	UK Biobank	NA
**Serum lipid**						
HDL cholesterol	310	403,943	European	167.36	UK Biobank	32203549
LDL cholesterol	148	440,546	European	185.77	UK Biobank	32203549
Total cholesterol	56	187,365	European	100.40	UK Biobank	NA
Triglycerides	269	441,016	European	164.41	UK Biobank	32203549
Apolipoprotein A-I	259	393,193	European	196.68	UK Biobank	32203549
Apolipoprotein B	172	439,214	European	225.16	UK Biobank	32203549
**Glucose**						
Type 2 diabetes	114	655,666	European	76.86	NA	30054458
Fasting insulin	38	151,013	European	52.47	NA	34059833
**Inflammatory factor**						
C-reactive protein	53	204,402	European	191.59	NA	30388399
**Sex hormones**						
Bioavailable testosterone	65	184,205	European	61.00	UK Biobank	NA
Oestradiol	12	163,985	European	79.30	UK Biobank	34255042
**Obesity traits**						
Body mass index	35	99,998	European	50.01	Within family GWAS Consortium	NA
Waist-to-hip ratio	28	212,244	European	40.94	GIANT	25673412
**Body composition**						
Arm fat mass (right)	255	331,226	European	57.21	UK Biobank	NA
Arm fat mass (left)	253	331,164	European	56.88	UK Biobank	NA
Leg fat mass (right)	267	331,293	European	57.82	UK Biobank	NA
Leg fat mass (left)	266	331,275	European	55.73	UK Biobank	NA
Whole body fat mass	261	330,762	European	56.68	UK Biobank	NA
Trunk fat mass	270	331,093	European	56.61	UK Biobank	NA
Whole body water mass	378	331,315	European	76.95	UK Biobank	NA
**Blood pressure**						
Hypertension	204	462,933	European	64.02	UK Biobank	NA
**Thyroid disease**						
Hyperthyroidism	6	72,167	European	47.82	NA	30367059
Hypothyroidism	78	337,159	European	71.08	UK Biobank	NA

### Data sources for OSA

To greatly avoid overlap with exposure GWASs, we extracted genetic instruments with OSA from the FinnGen study, which minimizes the incidence of Type I errors and enables less bias in the estimates. The FinnGen study involved 16,761 patients with OSA and 201,194 controls identified through the Finland nationwide health registries [31]. The diagnosis of OSA according to the International Classification of Diseases (Supplementary Table 1) was done, following subjective symptoms, clinical examination and sleep registration applying apnea-hypopnea index (AHI) ≥5/hour or respiratory event index (REI) ≥5/hour [31]. Age, sex, and ten genetic principal components were adjusted as covariates in the original GWAS study.

### Statistical analysis

For the main analyses, the inverse-variance weighted (IVW) models exhibits lead to precise causal estimates although it doesn’t correct for invalid instrument bias or pleiotropy [32]. The multiplicative random-effect IVW method is commonly reported as the best-performing method due to the advantage of maintaining asymptotic-free bias even when SNPs exhibit random horizontal pleiotropy effects [32]. Further, we verify the robustness of the findings and detect pleiotropy through several sensitivity analyses including weighted median [33], MR-Egger regression [34] and MR-PRESSO [35]. When at least 50% of the instrumental variables are valid, the weighted median approach was used to identify invalid instrument bias and deliver unbiased estimates [33]. MR-Egger regression was performed to detect and adjust for pleiotropy, although with relatively low precision [34]. The MR-PRESSO method is designed to check and exclude possible outliers that are potentially pleiotropy and then assess whether causal estimates have changed [35]. OR for causal estimates of fat mass (left and right) were combined using fixed-effects meta-analysis approaches [36]. Furthermore, we applied multivariable MR to extend the analysis of univariable MR, which allows using genetic variants of multiple potentially relevant exposures in a single model to infer the causal effect of each exposure on outcome [37]. As an essential complementary analysis strategy, multivariable MR was employed to analyze the effect of multiple exposures with common genetic predictors on outcome. We carried out multivariable MR to adjust BMI for significant results in univariable MR in order to prevent potential pleiotropy effects. Moreover, multivariable MR was used to distinguish the causal effects of fat mass and fat-free mass, even though the majority of genetic predictors of BMI influence both fat mass and fat-free mass. In the fixed-effect variance weighted analysis, Cochran’s Q statistics were used to assess the heterogeneity between instrumental variables. If Cochran’s Q indicated potential pleiotropy (*P* < 0.05), the random-effects model IVW approach was then utilized to draw inferences about causality [33]. We used a Bonferroni corrected significance level of *P* < 1.39 × 10^−3^ (0.05 divided by 34 risk factors). *P*-value between 1.22 × 10^−3^ and 0.05 was considered as suggestive associations. Findings are reported as OR and corresponding 95% CI. All statistical analyses were conducted in R 4.1.3 with R packages the Two Sample MR package [38], MRPRESSO [35], Mendelian Randomization package [39], and MVMR package [27].

## RESULTS

### Modifiable risk factors and OSA: Total effects

**Figure 2:**
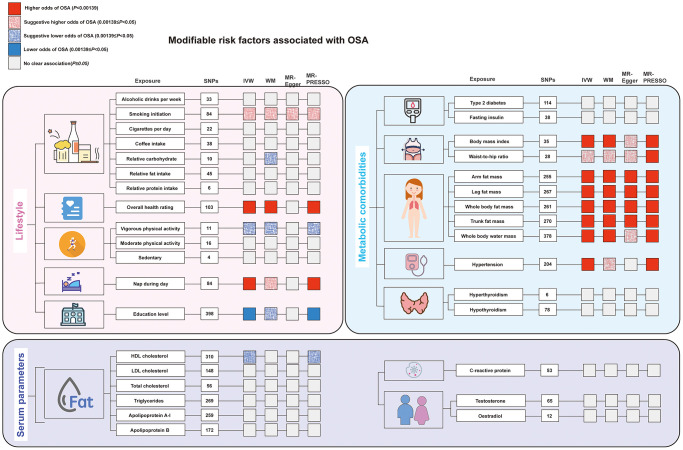
**The main results of the Mendelian randomization analysis of modifiable risk factors and OSA.** All results described here can be found in Figure 3 and Supplementary Table 3 in the Supplementary References. Abbreviations: HDL: high-density lipoprotein; LDL: low-density lipoprotein; MR-PRESSO: MR-pleiotropy residual sum and outlier; SNP: single nucleotide polymorphisms.

The causal relationship of 34 modifiable risk factors with OSA are demonstrated in Figure 2. Details of the instrumental variables used for the risk factors are presented in Table 1. The likelihood of a weak instrument bias is low because the F-statistics for genetic instruments for all risk factor traits are greater than 10 [40]. Supplementary Table 2 displays the proportion of cases and the total participant count within the outcome cohort, along with the detectable minimum odds ratio (OR) assuming 80% power for risk factors-OSA associations. The leave-one-SNP-out analysis conducted on meaningful risk factors did not identify any highly influential high leverage points (Supplementary Figures 1–5).

### Lifestyle factors

**Figure 3:**
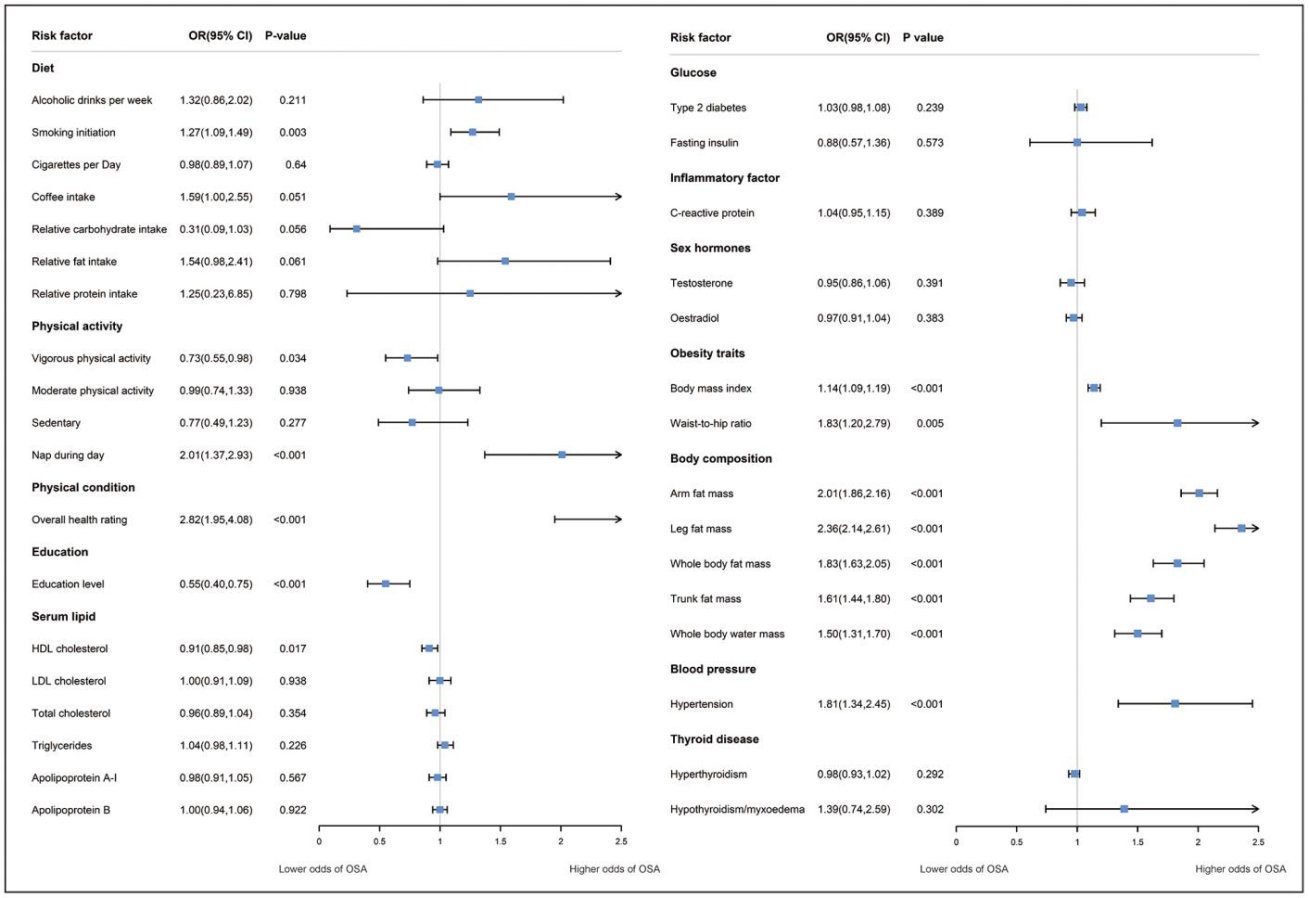
**The association between modifiable risk factors and OSA using the inverse-variance weighted method.** Odds ratios (ORs) represent the associations with OSA: one-SD increase in alcohol drink per week, coffee intake; relative carbohydrate intake, relative fat intake, vigorous/moderate physical activity, sedentary, c-reactive protein, HDL-cholesterol, LDL-cholesterol, total cholesterol, apolipoprotein A-I, apolipoprotein B, testosterone, oestradiol, body mass index, arm fat mass (left), leg fat mass (left), whole body fat mass, trunk fat mass, whole body water mass, fasting insulin, systolic blood pressure, diastolic blood pressure; one-SD increase in log-transformed odds in age of smoking initiation, overall health rating, age at menopause; one unit in log-transformed odds in insomnia, nap during day, sleep duration, education level, type 2 diabetes, hypothyroidism, hyperthyroidism, hypertension, polycystic ovarian syndrome, had menopause. Abbreviations: CI: confidence interval; HDL: high-density lipoprotein; LDL: low-density lipoprotein.

A significantly higher odds of OSA were observed for the modifiable risk factors including: genetic liability to overall health rating (OR 2.82; 95% CI: 1.95–4.08), nap during day (OR 2.01; 95% CI: 1.37–2.93), and genetically predicted education level (OR per 1 SD increase: 0.55; 95% CI: 0.40–0.75) was associated with a lower risk of OSA. Suggestive evidence was obtained for genetically predicted smoking initiation (OR 1.27; 95% CI: 1.09–1.49) with higher odds of OSA, vigorous physical activity (OR per 1 SD increase: 0.73, 95% CI: 0.55–0.98) with lower odds of OSA. No significant causal association was observed between genetically predicted alcoholic drinks per week, cigarettes per day, coffee intake, relative carbohydrate, fat, and protein intake, moderate physical activity, sedentary, insomnia, sleep duration and OSA (Figure 3).

For smoking initiation, overall health rating, and nap during day, MR-PRESSO identified two, four, and one outliers, respectively. (Supplementary Table 3). The causal association was generally consistent with the findings of the IVW analysis after potential pleiotropy and outliers identified by MR-PRESSO were removed. Genetically predicted smoking initiation (OR 1.21; 95% CI: 1.05–1.41), poor health condition (OR 2.40; 95% CI: 1.78–3.23), nap during day (OR 2.14; 95% CI: 1.48–3.10) for higher odds of OSA. Even though the heterogeneity test revealed heterogeneity in several risk factors, the IVW method’s estimates of causal effects under the random-effect model were confirmed.

### Serum parameters

Regarding serum parameters, MR analysis showed the protect effect of HDL-c on OSA risk (OR per 1 SD increase: 0.91, 95% CI: 0.95–0.98). No significant causal association was found between genetically driven LDL-c, total cholesterol, triglycerides, apolipoprotein A-I, apolipoprotein B, C-reactive protein, bioavailable testosterone, oestradiol and OSA (Figure 3). For HDL-c, both MR-Egger and MR-PESSO detected the presence of potential pleiotropy. After excluding potential pleiotropy and outliers, there is still suggestive evidence indicating a possible protective effect of HDL-c on OSA risk, although heterogeneity was observed (*P* = 0.015) (Supplementary Table 3).

### Metabolic comorbidities

We observed that all traits associated with obesity phenotype as well as body water mass were significantly associated with OSA risk: genetics predicted BMI (OR per 1-SD increase: 1.14; 95% CI: 1.09–1.19), waist-to-hip ratio (WHR) (OR per 1-SD increase: 1.83; 95% CI: 1.20–2.79), arm fat mass (OR per 1-SD increase: 2.01; 95% CI: 1.86–2.16), leg fat mass (OR per 1-SD increase: 2.36; 95% CI: 2.14–2.61), whole body fat mass (OR per 1-SD increase: 1.83; 95% CI: 1.63–2.05), trunk fat mass (OR per 1-SD increase: 1.61; 95% CI: 1.44–1.80), whole body water mass (OR per 1-SD increase: 1.50; 95% CI: 1.31–1.70). When estimating the correlation between obesity- and edema-related characteristics and OSA, we observed the presence of pleiotropy and heterogeneity in the tests of MR-PRESSO and Q statistics. However, the causal association remained robust across different MR assumptions after excluding the outliers identified by MR-PRESSO. Besides, there is evidence of a causal relationship between hypertension and increased risk of OSA (OR: 1.81; 95% CI: 1.34–2.45). No significant associations with OSA were observed for genetically predicted type 2 diabetes, fasting insulin, hyperthyroidism and hypothyroidism (Figure 3).

### Multivariable MR analysis of OSA: Direct effects

**Figure 4:**
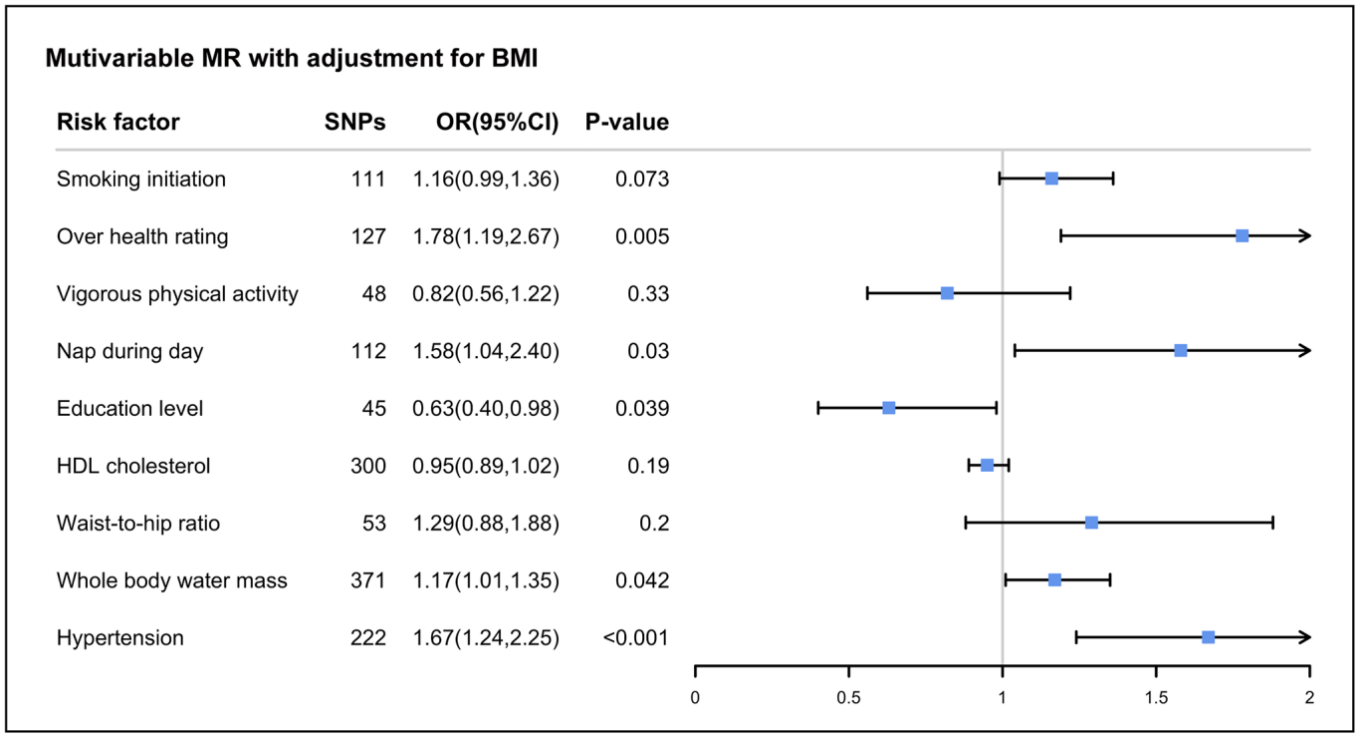
**The association between body mass index-adjusted modifiable risk factors and OSA by multivariable Mendelian randomization.** Abbreviations: OR: odds ratios; CI: confidence interval; HDL: high-density lipoprotein; LDL: low-density lipoprotein.

Given that obesity is prevalent in OSA patients, we applied multivariable MR to assess the direct effect of modifiable risk factors (including smoking initiation, overall health rating, vigorous physical activity, nap during day, education level, HDL-c, WHR, whole body water mass, hypertension, systolic blood pressure, diastolic blood pressure and age at menopause on the risk of OSA to adjust for the potential pleiotropy of BMI (Figure 4). The correlations for overall health rating (OR: 1.78; 95% CI: 1.19–2.67), nap during day (OR: 1.58; 95% CI: 1.04–2.40), education level (OR: 0.63; 95% CI: 0.40–0.98), whole body water mass (OR: 1.17; 95% CI: 1.01–1.35), and hypertension (OR: 1.67; 95% CI: 1.24–2.25) remained significant, which further confirms the robustness of the finding, although the effect becomes slightly weaker (Supplementary Table 4). Whereas, the significant association of smoking initiation, vigorous physical activity, HDL-c and WHR were fully attenuated in multivariable MR analysis (Supplementary Table 4). Therefore, the positive association of these risk factors we observed above may be dominated by the effect of BMI. The multivariable MR-Egger approach provided results consistent with the direction of the IVW analysis, and the intercept term did not imply the presence of pleiotropy, which further confirmed our findings (Supplementary Table 4). Almost all conditional F-statistics are greater than 10 or close to 10 (F_vigorous physical activity_ = 9.59), indicating that the majority of results ought not to suffer substantially from weak instrument bias.

It is significant to emphasize that the body composition measures for overall and obesity are interdependent. Given that most genetic predictions of fat mass and fat-free mass are influenced by BMI, we applied multivariable MR to adjust for fat-free mass in order to evaluate the direct effect of fat mass on OSA, details of the fat-free mass data sources are shown in Supplementary Table 5. Interestingly, we observed that fat mass in all parts of the body increased the risk of OSA, whereas arm fat-free mass may have a protective effect against OSA (Supplementary Table 6). Consistent results were also obtained with multivariable MR-Egger, and no pleiotropy was observed.

## DISCUSSION

To the best of our knowledge, the present study investigated the impact of the most widely modifiable risk factors on the risk of OSA based on a large-scale population. We observed that genetically predicted overall health rating, nap during day, BMI, body fat mass, body water mass, and hypertension were associated with higher odds of OSA, and a higher education level was associated with lower odds of OSA. For the other modifiable risk factors, there was not enough evidence for a causal association with OSA.

Considering the results validated by multiple sensitivity analyses, we observed that the risk causality between overall health rating, education level, nap during day, BMI, body fat mass, body water mass, hypertension and OSA are supposed to be robust. As shown in Supplementary Tables 6–9, there are a number of studies that have explored the association between lifestyle, serum parameters and metabolic comorbidities risk factors and OSA. Our findings support previously published observational studies [41–48], randomized controlled trials (RCT) [49–51] and MR studies [52–54]. Furthermore, compelling evidence for the association between overall obesity and OSA is provided by this study. A recently published MR study also suggested a causal relationship between BMI, WHR, diastolic blood pressure and OSA, which is in line with the basic picture we found [53]. Nevertheless, after conducting multivariable MR adjusted for BMI, the positive results for WHR in the current study vanished, which may suggest that BMI played a more significant effect than WHR. Also, we discovered that fat mass had a substantial negative impact on OSA and that both peripheral adiposity and abdominal adiposity were significant risk variables after controlling for fat-free mass using multivariable MR. Observational research on the potential link between physical activity and OSA risk have shown conflicting findings. One cross-sectional study including 9733 participants did not observe a protective effect of physical activity on OSA risk [55]. In addition, an RCT study showed a moderate treatment effect of 150 minutes/week of moderate-intensity aerobic exercise to reduce apnea-hypopnea index in sedentary overweight/obese adults, suggesting that exercise may be beneficial in the management of OSA [56]. In the total effect estimate of univariable MR analysis, vigorous physical activity was found to have a protective impact against OSA. Nevertheless, this benefit was attenuated after adjusting for BMI. Research has found that weight loss through diet or lifestyle interventions on top of continuous positive airway pressure (CPAP) can bring about clinically meaningful and sustainable improvements in OSA severity and comorbidities as well as health-related quality of life [57, 58]. Accordingly, for obese individuals, vigorous physical activity based on BMI management may lower the incidence of OSA. Furthermore, body fat distribution should also play a significant role in clinical decision-making in addition to BMI [59].

Numerous observational studies have supported the link between hypertension and OSA [60–63]. Yet, their temporal and causal relationship has not yet been conclusively established. A wealth of clinical studies and earlier MR investigations indicate that OSA may facilitate the development of hypertension [64–67]. Instead, limited research has focused on whether hypertension is also one of the risk factors for OSA [61, 63]. According to our research, genetically predicted hypertension may contribute to the development of OSA, which is in line with previous observational studies [63]. In addition, an observational study revealed that excessive daytime sleepiness may mediate part of the pathophysiological role in the link between hypertension and OSA, which is reinforced by our findings [68]. We discovered that the association between daytime napping and OSA was also causal, given that the majority of sensitivity analyses were consistent. Although there is limited research on the effect of daytime napping on the risk of OSA, regular long (>60 minutes) midday naps or a daily siesta should not be encouraged as there was a study suggesting a significant association with increased risk of coronary artery disease [69].

The current observational studies are inconsistent in conclusions as to whether smoking has adverse effects on OSA. Several cross-sectional studies did not observe an association between smoking (packs/year) and OSA [10, 70–73]. The present study observed suggestive evidence that smoking initiation was causally associated with an increased risk of OSA rather than cigarettes per day. Nevertheless, the association disappeared after adjusting for BMI, suggesting that this association can be influenced by BMI. Although there is insufficient evidence to support the risk of smoking for OSA, smoking was found to be frequent in severe OSA patients with more prevalent cardiovascular disease co-morbidities [71, 73].

We are currently inconclusive on the association of drinking, coffee intake, composition of diet structure, sedentary, insomnia, sleep duration, serum lipid, C-reactive protein, fasting insulin, type 2 diabetes, thyroid dysfunction, testosterone and oestradiol with OSA. Although the association between these risk factors [10, 74–85] and OSA was found in observational studies, it is not clear whether this is due to confounding or reverse causality bias in observational studies or the null associations finding in present study was a lack of statistical power due to the small phenotypic variance explained by the genetic instruments. Overall, no firm conclusions could be drawn about the causal associations between these risk factors and OSA in this MR study.

### Underlying mechanisms

OSA is characterized by intermittent pharyngeal obstruction occurring during sleep resulting in prolonged exposure to hypoxia, hypercapnia, increased sympathetic activity, oxidative stress, and systemic inflammation [86]. Modifiable risk factors can alter these pathophysiological processes through different pathways. Obesity, a major influence on OSA, can alter the anatomical structure and collapsibility of the airway as well as the regulation of the respiratory system [58]. As well, OSA exacerbates obesity through sympathetic hyperactivity and insulin resistance [42]. Increased visceral adipose tissue may be the cause of the secretion of inflammatory cytokines, which may lead to an altered sleep-wake rhythm [19]. Longer daytime naps may activate sympathetic nerves and cause large daytime fluctuations in blood pressure and heart rate [69]. Hypertension may cause salt and water retention, while fluid volume displacement from the legs to the neck at night is more likely to promote fluid accumulation in the neck, which may play a part in the development of upper airway obstruction [87, 88]. The favorable effect of higher levels of education on OSA risk may be that it leads to better knowledge and skills to make healthier and longer-term decisions regarding lifestyle and disease prevention, and more resources to maintain healthy lifestyles and access to medical care.

### Implications

Changing the lifestyle behaviors and modifiable risk factors identified in this study will potentially help prevent OSA and ultimately reduce the possible burden of cardiovascular disease associated with OSA. Each of the risk factors identified has its causal effect on OSA, and thus these are potential prevention targets. This study provides OSA prevention guidelines and government policymakers with information to improve public health and reduce educational inequalities in the population. Exposure or outcome GWAS requires larger sample sizes to be able to draw causal conclusions about certain risk factors, primarily lifestyle behaviors such as physical activity, dietary habits, and smoking.

### Strengths and limitations

There are several advantages of this study. First, we minimized potential bias from confounding and reverse causality by applying an MR design. Second, this study included the most risk factor phenotypes for OSA so far, and multiple sensitivity analyses were performed to validate that the instrumental variables met the plausibility of the hypothesis to increase the robustness of the results. Third, there was no sample overlap between exposure and outcome data sources to maintain the lowest possible type I error rate (Table 1).

However, several limitations should be realized. First, our inability to conclude certain risk factors may be due to the limited precision of the instrumental variables explained by the small variables. Second, as with all MR studies, the potential pleiotropy of genetic instruments is challenging [89]. However, we obtained robust results by applying multiple sensitivity analyses with different assumptions about the pleiotropy and after excluding MR-PRESSO to identify possible outliers. Third, despite employing the largest available GWASs for risk factors within our knowledge to report the powered correlations, not all 34 associations possess adequate statistical power. This could be attributed to the relatively modest portion of variances explained by instrumental variables for exposures. Notably null associations with low power should be interpreted cautiously to avoid false negative results. Moreover, most of the MR analyses with meaningful associations between risk factors and OSA had a statistical power of ≥80% in our study. Hence, it is imperative to conduct a subsequent GWAS with a more extensive sample size in order to validate and revise the conclusions drawn from this study. Fourth, our study was restricted to the European population, which limits the reduction of population stratification bias but may not be generalizable to other populations. Fifth, identifying SNPs associated with OSA severity and analyzing the relationship between risk factors and OSA severity would be an interesting direction in the future. However, we could not analyze this due to the lack of relevant SNP studies on OSA severity. Sixth, selection bias has an impact on the association between risk variables and OSA risk since a portion of symptomatic individuals are frequently not evaluated for polysomnography due to a lack of awareness of the disease. False negatives cause a bias in favor of the null hypothesis, which lowers power by underestimating the genuine causal effect.

## CONCLUSIONS

This study identified obesity, poor overall health rating, nap during day, high body fat mass, increased body water mass, low education level, and hypertension as causal risk factors for OSA, which prompted several key goals for the prevention of OSA and its related cardiovascular disease burden. Our work contributed to a clearer picture of the underlying risk factors for the development and progression of OSA. For other modifiable risk factors, there is insufficient evidence to draw conclusions about the causal association.

## Supplementary Materials

Supplementary Figures

Supplementary Tables 1-2 and 4-6

Supplementary Tables 3 and 7-9

Supplementary References
