# Supplementary material for: Association of modifiable risk factors with obstructive sleep apnea: a Mendelian randomization study
Source: Aging (Albany NY). 2023 Dec 11;15(23):14039–65. doi: 10.18632/aging.205288 (PMC10756101; doi:10.18632/aging.205288)
Supplement: Supplementary Figures [file aging-15-205288-s001.pdf]

SUPPLEMENTARY FIGURES

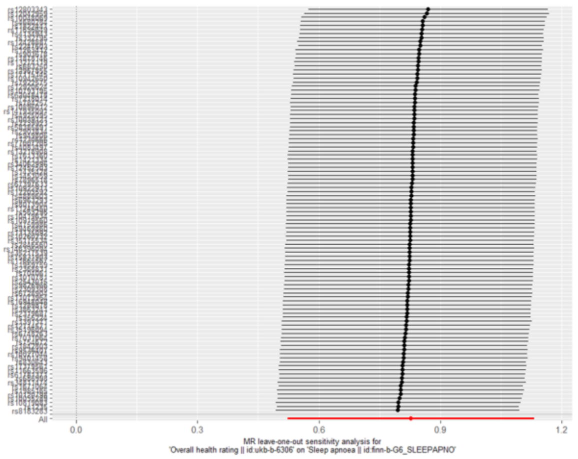

Overall health rating

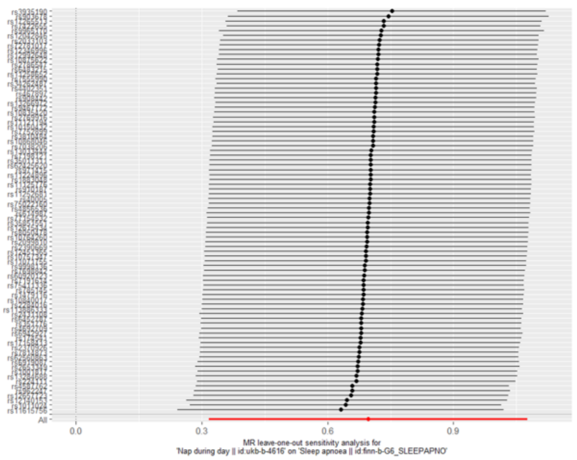

Nap during day

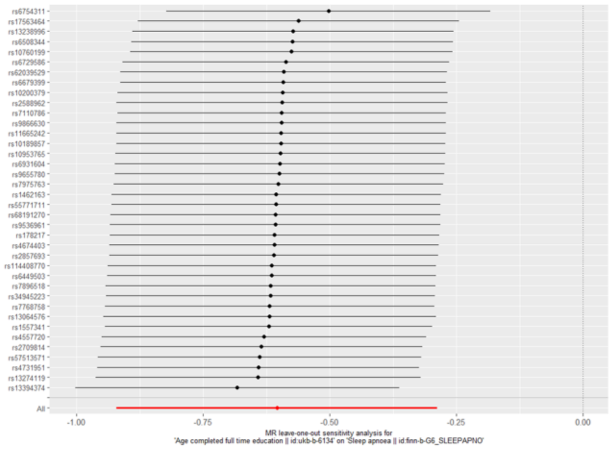

Education level

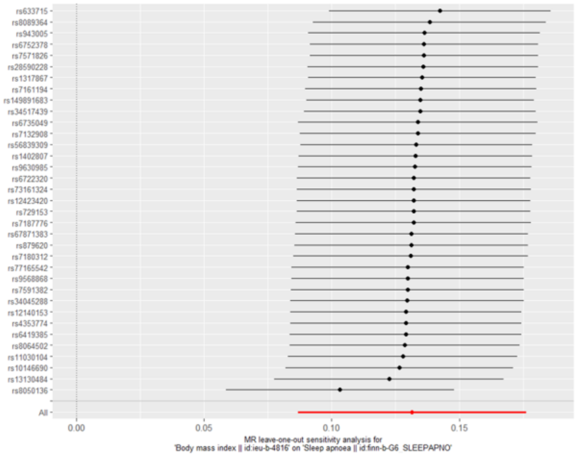

BMI

Supplementary Figure 1. Leave-one-out analyses for SNPs associated with risk factors (overall health rating/nap during day/education level/BMI) on OSA.

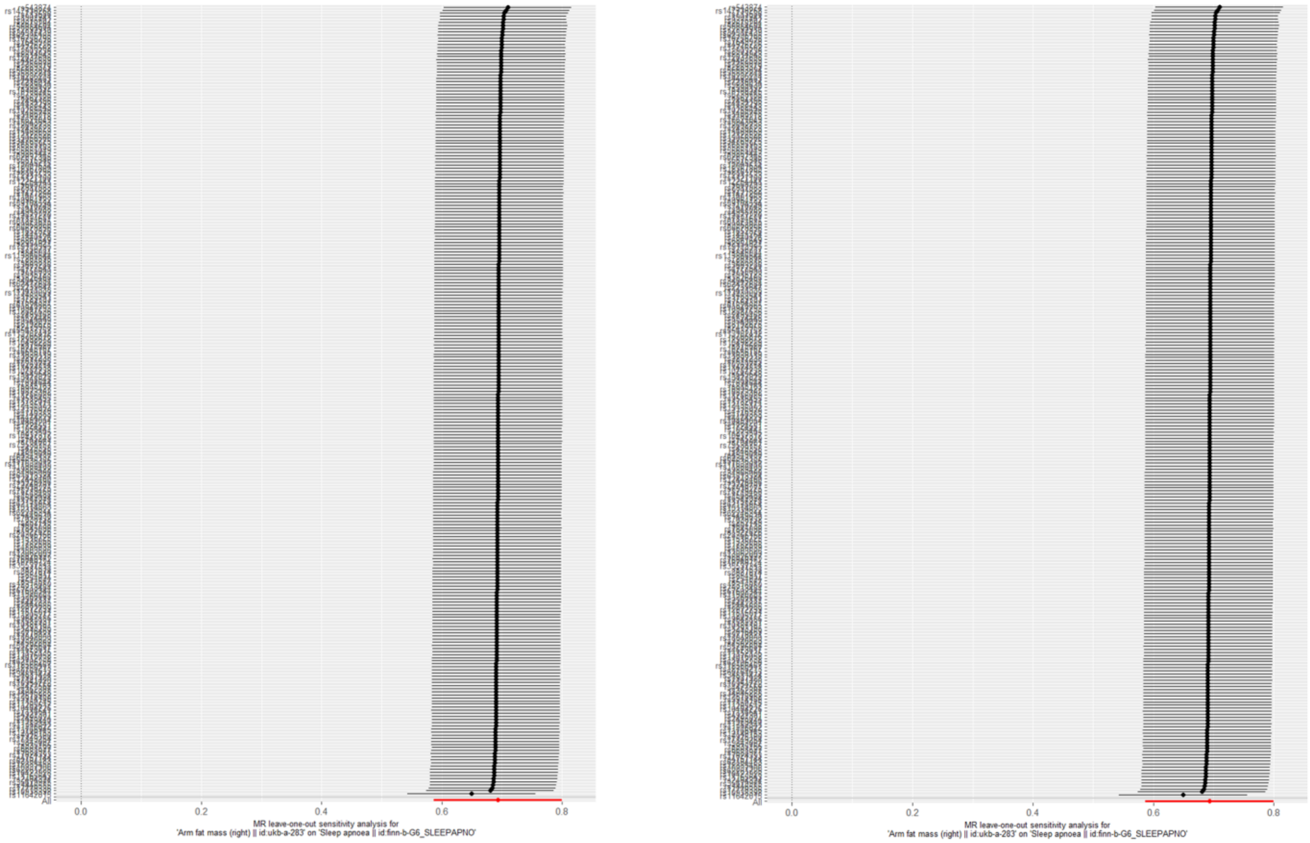

Arm fat mass (left) Arm fat mass (right)

Supplementary Figure 2. Leave-one-out analyses for SNPs associated with risk factors (arm fat mass left/right) on OSA.

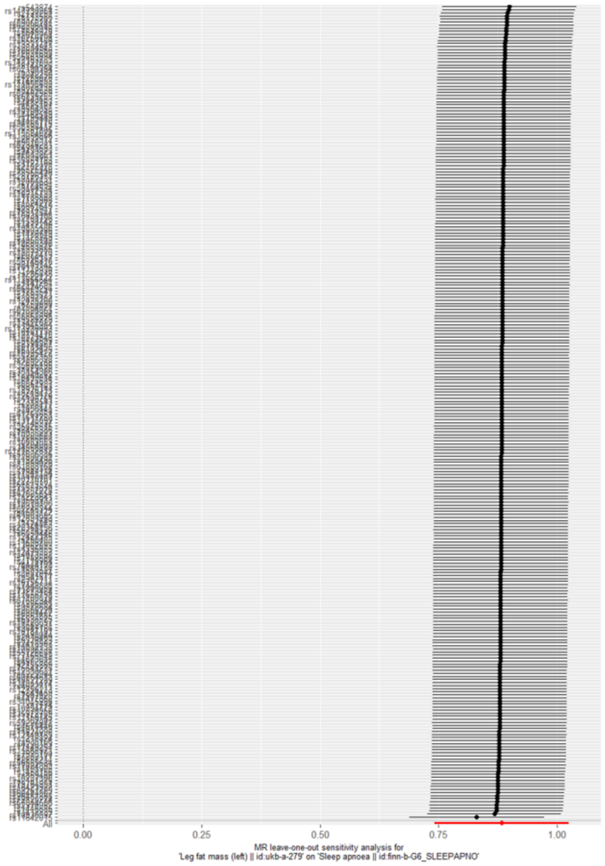

Leg fat mass (left)

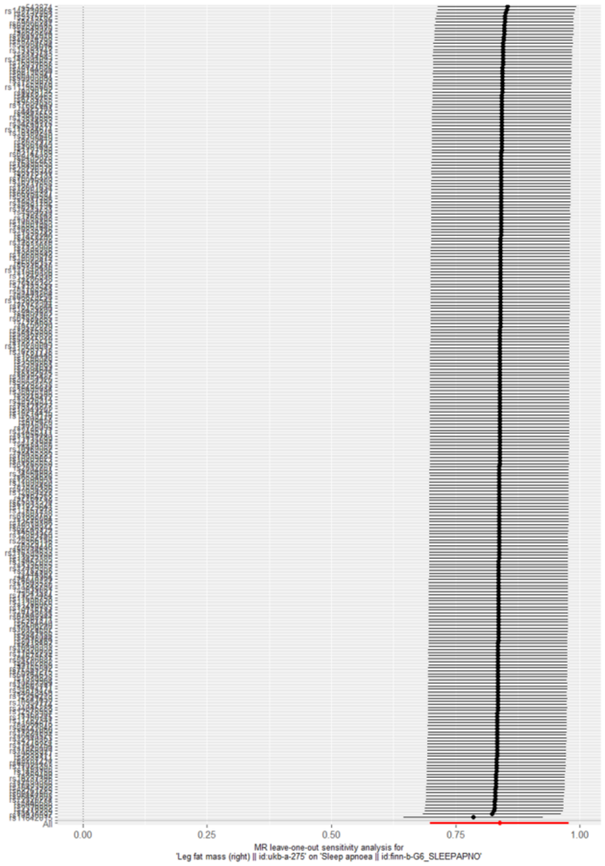

Leg fat mass (right)

Supplementary Figure 3. Leave-one-out analyses for SNPs associated with risk factors (leg fat mass left/right) on OSA.

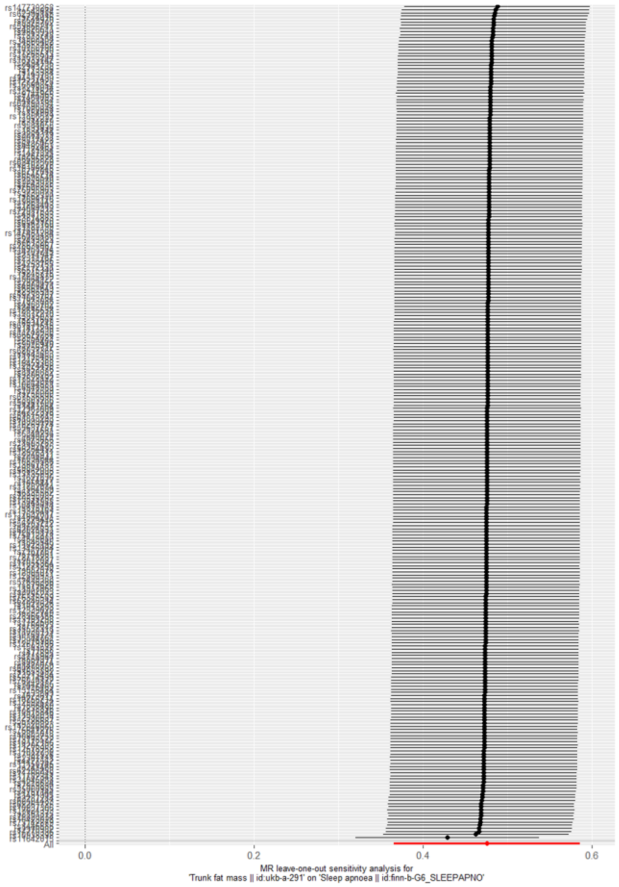

Trunk fat mass

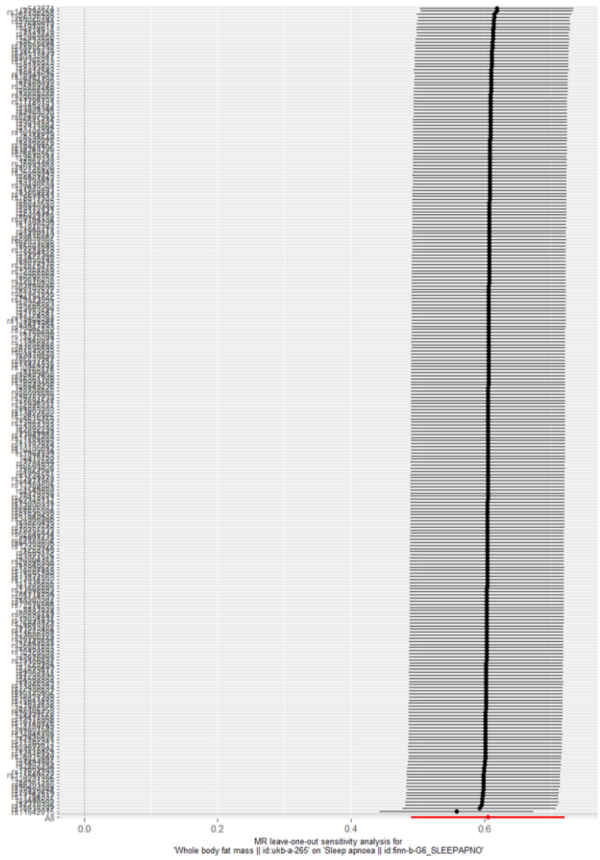

Whole body fat mass

Supplementary Figure 4. Leave-one-out analyses for SNPs associated with risk factors (trunk fat mass/whole body fat mass) on OSA.

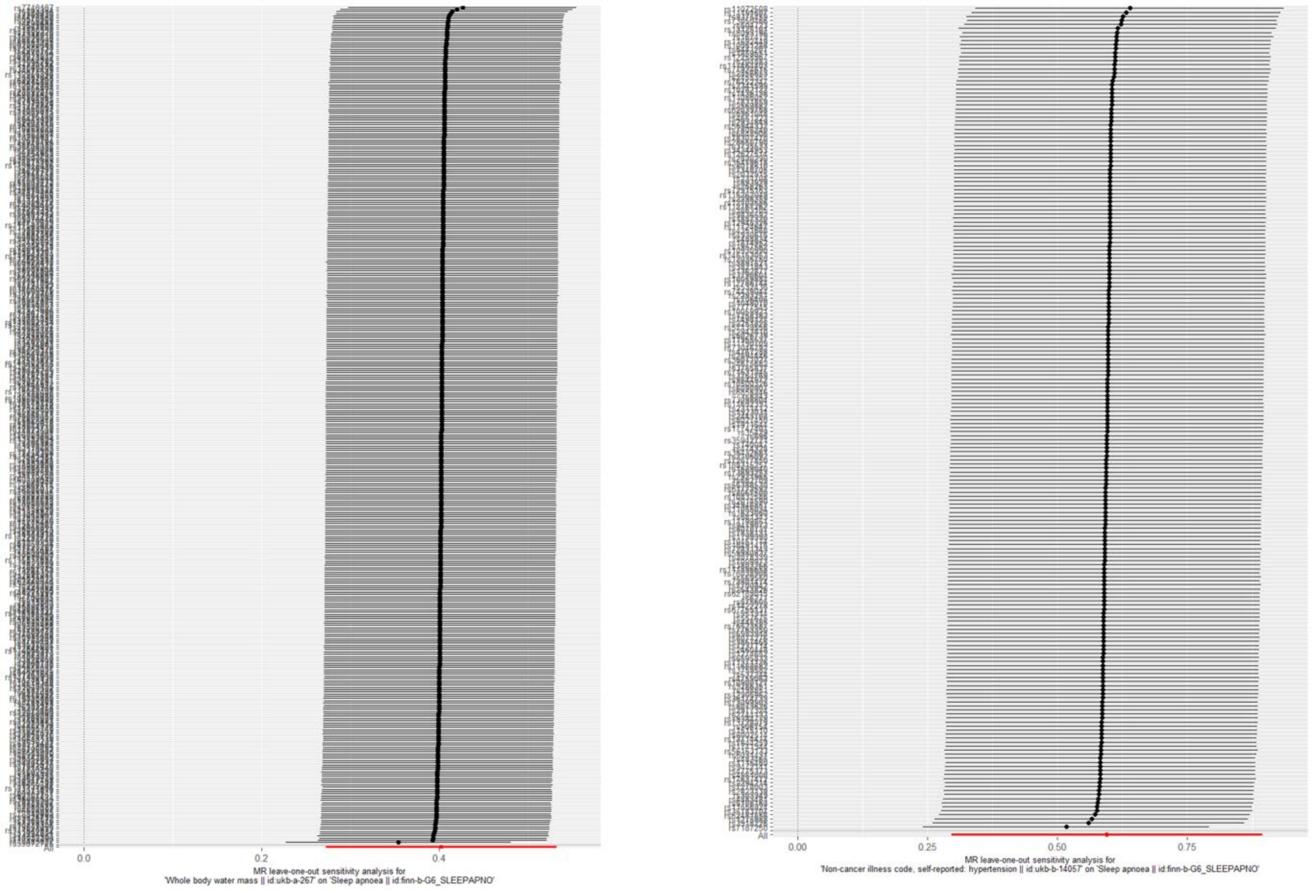

Whole body water mass

Hypertension

Supplementary Figure 5. Leave-one-out analyses for SNPs associated with meaningful risk factors (whole body water mass/hypertension) on OSA.
