# Supplementary material for: Association of modifiable risk factors with obstructive sleep apnea: a Mendelian randomization study
Source: Aging (Albany NY). 2023 Dec 11;15(23):14039–65. doi: 10.18632/aging.205288 (PMC10756101; doi:10.18632/aging.205288)
Supplement: Supplementary Tables 1-2 and 4-6 [file aging-15-205288-s002.pdf]

SUPPLEMENTARY TABLES

Supplementary Table 1. Overview of the used international classification of diseases (ICD) diagnosis codes to define cases with OSA and controls in the second release of the FinnGen Study.

		Cases with OSA<br>Participants with at least one of the following diagnoses according to the electronic health registries	Controls<br>Participants without any of the following diagnoses according to the electronic health registries
Diagnostic classification codes	ICD-10	G47.3	G47.4 | G47
	ICD-9	3472	347 [0–1] | 3478 | 3074
	ICD-8		34700

Supplementary Table 2. The statistical power of the univariable MR at 5% type I error.

Risk factor	Variance explained (R^2) by the instrument (%)	Outcome sample size	Ratio of cases to controls	Minimum causal effect (Odds Ratio) detectable with 80% power	
**Diet**					
Alcoholic drinks per week	0.50	217955	0.083	≤0.73	≥1.38
Smoking initiation	0.61	217955	0.083	≤0.75	≥1.29
Cigarettes per day	0.91	217955	0.083	≤0.79	≥1.33
Coffee intake	0.68	217955	0.083	≤0.76	≥1.31
Relative carbohydrate intake	0.16	217955	0.083	≤0.57	≥1.76
Relative fat intake	0.48	217955	0.083	≤0.72	≥1.33
Relative protein intake	0.12	217955	0.083	≤0.52	≥1.92
**Physical activity**					
Number of days/week of vigorous physical activity 10+ minutes	0.10	217955	0.083	≤0.49	≥2.04
Number of days/week of moderate physical activity 10+ minutes	0.13	217955	0.083	≤0.54	≥1.87
Sedentary	0.16	217955	0.083	≤0.57	≥1.76
Nap during day	0.88	217955	0.083	≤0.79	≥1.27
**Physical condition**					
Overall health rating	0.93	217955	0.083	≤0.79	≥1.26
**Education**					
Education level	0.48	217955	0.083	≤0.72	≥1.39
**Serum lipid**					
HDL cholesterol	11.61	217955	0.083	≤0.94	≥1.07
LDL cholesterol	6.06	217955	0.083	≤0.91	≥1.10
Total cholesterol	2.93	217955	0.083	≤0.88	≥1.14
Triglycerides	9.55	217955	0.083	≤0.93	≥1.08
Apolipoprotein A-I	10.49	217955	0.083	≤0.93	≥1.07
Apolipoprotein B	8.12	217955	0.083	≤0.92	≥1.08
**Glucose**					
Type 2 diabetes	1.41	217955	0.083	≤0.83	≥1.21
Fasting insulin	1.30	217955	0.083	≤0.82	≥1.22
**Inflammatory factor**					
C-reactive protein	4.76	217955	0.083	≤0.90	≥1.11
**Sex hormones**					
Bioavailable testosterone	3.27	217955	0.083	≤0.88	≥1.13

Oestradiol	0.58	217955	0.083	≤0.74	≥1.35
**Obesity traits**					
Body mass index	1.62	217955	0.083	≤0.84	≥1.19
Waist-to-hip ratio	0.68	217955	0.083	≤0.76	≥1.31
**Body composition**					
Arm fat mass (right)	4.54	217955	0.083	≤0.90	≥1.11
Arm fat mass (left)	4.52	217955	0.083	≤0.90	≥1.11
Leg fat mass (right)	4.62	217955	0.083	≤0.90	≥1.11
Leg fat mass (left)	4.65	217955	0.083	≤0.90	≥1.11
Whole body fat mass	4.62	217955	0.083	≤0.90	≥1.11
Trunk fat mass	4.68	217955	0.083	≤0.90	≥1.11
Whole body water mass	8.65	217955	0.083	≤0.93	≥1.08
**Blood pressure**					
Hypertension	2.94	217955	0.083	≤0.88	≥1.14
**Thyroid disease**					
Hyperthyroidism	0.40	217955	0.083	≤0.70	≥1.43
Hypothyroidism	1.64	217955	0.083	≤0.84	≥1.19

The statistical power to assess causal effect was calculated by an online tool (https://sb452.shinyapps.io/power/). According to variance explained (R^2) of the instrument, sample size, and the ratio of cases to controls, we calculate the minimum negative/positive causal effect detectable with 80% power.

Supplementary Table 4. The association between body mass index-adjusted modifiable risk factors and OSA by multivariable Mendelian randomization.

Risk factor	SNPs	IVW	*P*	MR-Egger	*P*	Egger-intercept	Conditional F-statistics
		OR (95% CI)		OR (95% CI)			
Smoking initiation	111	1.16 (0.99,1.36)	0.073	1.15 (0.99,1.32)	0.086	0.507	30.28
Over health rating	127	1.78 (1.19,2.67)	**0.005**	1.27 (0.74,1.79)	0.375	0.051	17.94
Vigorous physical activity	48	0.82 (0.56,1.22)	0.330	0.83 (0.44,1.22)	0.360	0.274	9.59
Nap during day	112	1.58 (1.04,2.40)	**0.030**	1.61 (1.19,2.03)	**0.025**	0.247	33.77
Education level	45	0.63 (0.40,0.98)	**0.039**	0.62 (0.14,1.10)	**0.048**	0.858	26.66
HDL cholesterol	300	0.95 (0.89,1.02)	0.190	0.95 (0.88,1.02)	0.190	0.898	123.88
Waist-to-hip ratio	53	1.29 (0.88,1.88)	0.200	1.28 (0.89,1.67)	0.210	0.856	14.99
Whole body water mass	371	1.17 (1.01,1.35)	**0.042**	1.33 (1.11,1.54)	**0.038**	0.396	11.50
Hypertension	222	1.67 (1.24,2.25)	**<0.001**	1.66 (1.36,1.95)	**0.001**	0.139	52.83

Abbreviations: SNP: single nucleotide polymorphisms; IVW: inverse variance weighted; OR: odds ratio; CI: confidence interval.

Supplementary Table 5. Sources and characteristics of fat-free mass data for multivariable Mendelian randomization analysis.

Exposure	Sample	Ethnicity	Consortium
Arm fat-free mass (right)	331,221	European	UK Biobank
Arm fat-free mass (left)	331,159	European	UK Biobank
Leg fat-free mass (right)	331,285	European	UK Biobank
Leg fat-free mass (left)	331,258	European	UK Biobank
Whole body fat-free mass	331,291	European	UK Biobank
Trunk fat-free mass	331,030	European	UK Biobank

Supplementary Table 6. The association between fat-free mass-adjusted body components and OSA by multivariable Mendelian randomization.

Exposure	SNPs	IVW	*P*	MR-Egger	*P*	Egger-intercept
		OR (95%CI)		OR (95%CI)		
Arm fat mass (right)	429	2.29 (1.89,2.77)	<0.001	2.30 (2.08,2.53)	<0.001	0.92
Arm fat-free mass (right)		0.77 (0.62,0.97)	0.025	0.78 (0.51,1.04)	0.063	
Arm fat mass (left)	442	2.32 (1.92,2.81)	<0.001	2.23 (2.01,2.45)	<0.001	0.452
Arm fat-free mass (left)		0.79 (0.63,0.98)	0.033	0.74 (0.48,1.01)	0.028	
Leg fat mass (right)	461	2.44 (1.92,3.09)	<0.001	2.43 (2.14,2.72)	<0.001	0.969
Leg fat-free mass (right)		0.92 (0.74,1.14)	0.440	0.92 (0.66,1.17)	0.492	
Leg fat mass (left)	477	2.45 (1.89,3.17)	<0.001	2.61 (2.30,2.92)	<0.001	0.447
Leg fat-free mass (left)		0.89 (0.70,1.13)	0.350	0.94 (0.66,1.22)	0.679	
Whole body fat mass	472	2.19 (1.80,2.67)	<0.001	2.19 (1.96,2.42)	<0.001	0.976
Whole body fat-free mass		0.82 (0.66,1.01)	0.620	0.82 (0.57,1.07)	0.112	
Trunk fat mass	463	1.85 (1.55,2.21)	0.001	1.91 (1.69,2.13)	<0.001	0.626
Trunk fat-free mass		0.90 (0.74,1.08)	0.240	0.91 (0.71,1.12)	0.382	

Abbreviations: SNP: single nucleotide polymorphisms; IVW: inverse variance weighted; OR: odds ratio; CI: confidence interval.
