# Supplementary material for: Association of modifiable risk factors with obstructive sleep apnea: a Mendelian randomization study
Source: Aging (Albany NY). 2023 Dec 11;15(23):14039–65. doi: 10.18632/aging.205288 (PMC10756101; doi:10.18632/aging.205288)
Supplement: Supplementary Tables 3 and 7-9 [file aging-15-205288-s003.docx]

**Supplementary Table 3. Main analysis and sensitivity analyses of the association of modifiable risk factors and lifestyle behaviors with OSA in FinnGen.**

|  |  | **Main analysis** | | **Sensitivity analysis** | | | | | | | | | |
| --- | --- | --- | --- | --- | --- | --- | --- | --- | --- | --- | --- | --- | --- |
| **Risk factor** | **SNPs** | **IVW** |  | **Weighted median** | | **MR Egger** |  | **MR-PRESSO** |  |  |  | **Cochrane's Q** | **Cochrane's Q Pvalue** |
|  |  | **OR (95%CI)** | **P** | **OR (95%CI)** | **P** | **OR (95%CI)** | **P** | **Outliers/SNPs** | **OR (95%CI)** | **P** | **P*_intercept_*** |  |  |
| **Diet** |  |  |  |  |  |  |  |  |  |  |  |  |  |
| Alcoholic drinks per week | 33 | 1.32(0.86,2.02) | 0.211 | 1.34(0.81,2.21) | 0.260 | 2.14(0.81,5.65) | 0.136 | 0/33 | 1.32(0.86,2.02) | 0.211 | 0.285 | 63.97 | <0.001 |
| Smoking initiation | 84 | 1.27(1.09,1.49) | **0.003** | 1.26(1.05,1.52) | **0.014** | 2.48(1.13,5.43) | **0.025** | 2/84 | 1.21(1.05,1.41) | **0.010** | 0.116 | 154.69 | <0.001 |
| Cigarettes per day | 22 | 0.98(0.89,1.07) | 0.640 | 0.96(0.85,1.08) | 0.463 | 0.93(0.78,1.11) | 0.424 | 0/22 | 0.98(0.89,1.07) | 0.640 | 0.492 | 24.99 | 0.248 |
| Coffee intake | 38 | 1.59(1.00,2.55) | 0.051 | 1.34(1.19,2.12) | 0.196 | 1.76(0.68,4.57) | 0.254 | 1/38 | 1.25(0.91,1.74) | 0.173 | 0.698 | 43.89 | 0.172 |
| Relative carbohydrate intake | 10 | 0.31(0.09,1.03) | 0.056 | 0.47(0.22,1.00) | **0.049** | 7.57(0.00,12851.27) | 0.608 | 3/10 | 1.25(0.93,1.68) | 0.133 | 0.585 | 10.15 | 0.118 |
| Relative fat intake | 45 | 1.54(0.98,2.41) | 0.061 | 1.09(0.71,1.69) | 0.692 | 0.84(0.25,2.78) | 0.779 | 5/45 | 1.12(0.78,1.59) | 0.549 | 0.196 | 59.29 | 0.032 |
| Relative protein intake | 6 | 1.25(0.23,6.85) | 0.798 | 0.82(0.44,1.54) | 0.539 | 0.05(0.00,47.10) | 0.444 | 1/6 | 0.63(0.39,1.04) | 0.070 | 0.640 | 2.47 | 0.650 |
| **Physical activity** |  |  |  |  |  |  |  |  |  |  |  |  |  |
| Number of days/week of vigorous physical activity 10+ minutes | 11 | 0.73(0.55,0.98) | **0.034** | 0.65(0.44,0.97) | **0.036** | 0.92(0.08,11.16) | 0.951 | 0/11 | 0.73(0.55,0.98) | **0.034** | 0.860 | 10.78 | 0.375 |
| Number of days/week of moderate physical activity 10+ minutes | 16 | 0.99(0.74,1.33) | 0.938 | 0.94(0.68,1.29) | 0.690 | 2.44(0.35,17.09) | 0.384 | 0/16 | 0.99(0.74,1.33) | 0.938 | 0.372 | 30.46 | 0.010 |
| Sedentary | 4 | 0.77(0.49,1.23) | 0.277 | 0.67(0.37,1.21) | 0.182 | 7.49(0.82,68.60) | 0.217 | 0/4 | 0.77(,0.49,1.23) | 0.277 | 0.366 | 4.73 | 0.192 |
| Nap during day | 84 | 2.01(1.37,2.93) | **<0.001** | 2.09(1.27,3.44) | **0.004** | 4.02(1.00,16.27) | 0.054 | 0/84 | 2.01(1.37,2.93) | **<0.001** | 0.409 | 114.90 | 0.012 |
| **Physical condition** |  |  |  |  |  |  |  |  |  |  |  |  |  |
| Overall health rating | 103 | 2.82(1.95,4.08) | **<0.001** | 2.55(1.72,3.79) | **<0.001** | 1.00(0.16,6.17) | 0.997 | 4/103 | 2.40(1.78,3.23) | **<0.001** | 0.217 | 135.97 | 0.008 |
| **Education** |  |  |  |  |  |  |  |  |  |  |  |  |  |
| Education level | 38 | 0.55(0.40,0.75) | **<0.001** | 0.60(0.39,0.91) | **0.017** | 1.92(0.46,8.02) | 0.376 | 0/38 | 0.55(0.40,0.75) | **<0.001** | 0.085 | 37.59 | 0.442 |
| **Serum lipid** |  |  |  |  |  |  |  |  |  |  |  |  |  |
| HDL cholesterol | 310 | 0.91(0.85,0.98) | **0.017** | 0.90(0.81,1.01) | 0.074 | 1.03(0.92,1.16) | 0.574 | 4/310 | 0.93(0.87,0.99) | **0.023** | 0.022 | 385.68 | 0.001 |
| LDL cholesterol | 148 | 1.00(0.91,1.09) | 0.938 | 0.96(0.86,1.07) | 0.461 | 1.00(0.88,1.15) | 0.952 | 2/148 | 1.01(0.93,1.08) | 0.881 | 0.556 | 170.02 | 0.170 |
| Total cholesterol | 56 | 0.96(0.89,1.04) | 0.354 | 0.92(0.82,1.03) | 0.159 | 1.00(0.86,1.16) | 0.979 | 0/56 | 0.96(0.89,1.04) | 0.354 | 0.588 | 49.34 | 0.690 |
| Triglycerides | 269 | 1.04(0.98,1.11) | 0.226 | 0.98(0.89,1.08) | 0.718 | 0.99(0.90,1.09) | 0.825 | 0/269 | 1.04(0.98,1.11) | 0.226 | 0.152 | 316.60 | 0.023 |
| Apolipoprotein A-I | 259 | 0.98(0.91,1.05) | 0.567 | 0.95(0.86,1.06) | 0.380 | 0.99(0.89,1.12) | 0.927 | 1/259 | 0.99(0.92,1.06) | 0.713 | 0.929 | 335.44 | 0.005 |
| Apolipoprotein B | 172 | 1.00(0.94,1.06) | 0.922 | 0.99(0.90,1.10) | 0.860 | 1.00(0.91,1.09) | 0.998 | 0/172 | 1.00(0.94,1.06) | 0.922 | 0.924 | 194.27 | 0.105 |
| **Glucose** |  |  |  |  |  |  |  |  |  |  |  |  |  |
| Type 2 diabetes | 114 | 1.03(0.98,1.08) | 0.239 | 0.97(0.91,1.04) | 0.427 | 0.98(0.87,1.10) | 0.722 | 2/114 | 1.00(0.97,1.04) | 0.914 | 0.135 | 131.23 | 0.092 |
| Fasting insulin | 38 | 0.88(0.57,1.36) | 0.573 | 1.00(0.61,1.62) | 0.990 | 0.84(0.20,3.42) | 0.812 | 1/38 | 0.96(0.31,1.45) | 0.840 | 0.965 | 72.63 | <0.001 |
| **Inflammatory factor** |  |  |  |  |  |  |  |  |  |  |  |  |  |
| C-reactive protein | 53 | 1.04(0.95,1.15) | 0.389 | 1.02(0.93,1.11) | 0.738 | 0.99(0.85,1.14) | 0.856 | 1/53 | 1.02(0.95,1.10) | 0.560 | 0.204 | 69.82 | 0.044 |
| **Sex hormones** |  |  |  |  |  |  |  |  |  |  |  |  |  |
| Bioavailable testosterone | 65 | 0.95(0.86,1.06) | 0.391 | 0.93(0.79,1.10) | 0.419 | 1.03(0.80,1.32) | 0.827 | 0/65 | 0.95(0.86,1.06) | 0.391 | 0.325 | 69.54 | 0.296 |
| Oestradiol | 12 | 0.97(0.91,1.04) | 0.383 | 0.92(0.85,1.01) | 0.089 | 0.89(0.70,1.14) | 0.392 | 0/12 | 0.97(0.91,1.04) | 0.383 | 0.107 | 16.93 | 0.110 |
| **Obesity traits** |  |  |  |  |  |  |  |  |  |  |  |  |  |
| Body mass index | 35 | 1.14(1.09,1.19) | **<0.001** | 1.11(1.05,1.17) | **<0.001** | 1.22(1.08,1.38) | **0.003** | 2/35 | 1.12(1.07,1.17) | **<0.001** | 0.816 | 57.56 | 0.004 |
| Waist-to-hip ratio | 28 | 1.83(1.20,2.79) | **0.005** | 1.57(1.11,2.22) | **0.011** | 15.77(2.78,89.39) | **0.004** | 6/28 | 1.56(1.21,2.01) | **<0.001** | 0.671 | 24.46 | 0.271 |
| **Body Composition** |  |  |  |  |  |  |  |  |  |  |  |  |  |
| Arm fat mass (right) | 255 | 2.00(1.80,2.23) | **<0.001** | 1.86(1.62,2.14) | **<0.001** | 2.08(1.52,2.85) | **<0.001** | 4/255 | 1.98(1.79,2.19) | **<0.001** | 0.217 | 317.42 | <0.001 |
| Arm fat mass (left) | 253 | 2.01(1.81,2.24) | **<0.001** | 1.84(1.58,2.13) | **<0.001** | 1.89(1.38,2.59) | **<0.001** | 4/253 | 1.95(1.76,2.17) | **<0.001** | 0.057 | 396.30 | <0.001 |
| Leg fat mass (right) | 267 | 2.31(2.01,2.66) | **<0.001** | 2.09(1.76,2.49) | **<0.001** | 2.46(1.59,3.80) | **<0.001** | 10/267 | 2.22(1.95,2.54) | **<0.001** | 0.251 | 489.40 | <0.001 |
| Leg fat mass (left) | 266 | 2.42(2.10,2.78) | **<0.001** | 2.23(1.88,2.64) | **<0.001** | 2.75(1.79,4.22) | **<0.001** | 4/266 | 2.33(2.03,2.67) | **<0.001** | 0.548 | 483.71 | <0.001 |
| Whole body fat mass | 261 | 1.83(1.63,2.05) | **<0.001** | 1.64(1.43,1.89) | **<0.001** | 2.08(1.47,2.94) | **<0.001** | 11/261 | 1.74(1.57,1.93) | **<0.001** | 0.462 | 478.04 | <0.001 |
| Trunk fat mass | 270 | 1.61(1.44,1.80) | **<0.001** | 1.48(1.29,1.69) | **<0.001** | 1.96(1.40,2.76) | **<0.001** | 11/270 | 1.50(1.36,1.65) | **<0.001** | 0.660 | 495.23 | <0.001 |
| Whole body water mass | 378 | 1.50(1.31,1.70) | **<0.001** | 1.37(1.16,1.61) | **<0.001** | 1.12(0.82,1.53) | 0.492 | 10/378 | 1.39(1.24,1.56) | **<0.001** | 0.035 | 678.48 | <0.001 |
| **Blood pressure** |  |  |  |  |  |  |  |  |  |  |  |  |  |
| Hypertension | 204 | 1.81(1.34,2.45) | **<0.001** | 1.47(1.01,2.13) | **0.044** | 1.13(0.49,2.57) | 0.779 | 2/204 | 1.75(1.33,2.23) | **<0.001** | 0.222 | 242.35 | 0.008 |
| **Thyroid disease and related trait** |  |  |  |  |  |  |  |  |  |  |  |  |  |
| Hyperthyroidism | 6 | 0.98(0.93,1.02) | 0.292 | 0.97(0.92,1.03) | 0.324 | 0.96(0.78,1.18) | 0.720 | 0/6 | 0.98(0.93,1.02) | 0.292 | 0.881 | 2.43 | 0.788 |
| Hypothyroidism/myxoedema | 78 | 1.39(0.74,2.59) | 0.302 | 2.00(0.72,5.56) | 0.183 | 1.04(0.27,4.03) | 0.959 | 0/78 | 1.39(0.74,2.59) | 0.302 | 0.636 | 69.58 | 0.714 |

SNP, single nucleotide polymorphisms; IVW, inverse variance weighted; WM, weighted median; MR-PRESSO, MR-pleiotropy residual sum and outlier; OR, odds ratio; CI, confidence interval.

**Supplementary Table 7. Summary of the current studies examining the associations of lifestyle risk factors with OSA.**

| **Study design** | **Year** | **Country** | **Sample size** | **Status** | **Sleep apnea** | **Age** | **Follow-up duration** | **Intervention** | **Outcome** | **References** |
| --- | --- | --- | --- | --- | --- | --- | --- | --- | --- | --- |
| **Clinical trial study** | 2005 | Greece | 83 | High-intelligence group (IQ ≥90ile percentile) (n=39) and a normal-intelligence group (50 ≤ IQ < 90%ile) (n=44) | polysomnographic diagnosis of OSA | Case 47.3± 7.9 years  Control 49.3± 2.9 years | 13.7 ± 0.8 months | CPAP to 4.5 h of use per night at least 1 year | OSA patients with normal intelligence showed neuropsychological deficits regarding reaction time. These deficits were fully corrected after prolonged therapy with CPAP. | [1**]** |
| **Cross-sectional study** | 2021 | United States | 109 | Wisconsin Card Sorting Test, WAIS-III digit span and block design, semantic and phonemic fluency tests, psychomotor vigilance task. | OSA (mean AHI 32.58) | Case 54.82±8.56 years  Control 56.60±12.56 years | - | - | In semantic fluency and visuospatial ability tasks, patients with higher education performed better than patients with lower education and controls with lower education. | [2] |
| **multicenter cross-sectional study** | 2022 | Saudi Arabia. | 150 | Children with sickle cell disease (SCD) | OSA diagnosed with Pediatric Sleep Questionnaire | 2 -8 years | - | - | Education level was associated with OSA in the bivariate analysis | [3] |
| **Cross-sectional study** | 2000 | USA | 435 | Underwent health status assessment, including the Medical Outcomes Study SF-36 Health Survey and the Pittsburgh Sleep Quality Index. | RDI >10 | 45.7±11.9 years | - | - | Sleep disturbances demonstrate significant decrements in general and sleep-specific health status | [4] |
| **Cross-sectional study** | 2021 | China | 9733 | Assessing three dimensions of physical activity, including LTPA, transport activity, and occupational activity | OSA was ascertained by using Berlin Questionnaire | Case 57.48±9.28 years  Control 55.67±9.97 years | - | - | leisure-time physical activity, transport activity, and occupational activity, were not associated with any risk of OSA. | [5] |
| **RCT** | 2011 | USA | 43 | Sedentary and overweight/obese adults | moderate-severity untreated OSA (AHI ≥ 15) | 46.9 ±1.2 | 12 weeks | 4 times/week for 12 weeks and performed 150 min/week of moderate-intensity aerobic activity, followed by resistance training twice/week. | Exercise training had moderate treatment efficacy for the reduction of AHI in sedentary overweight/obese adults | [6] |
| **Cross-sectional study** | 2019 | Taiwan | 733 | Current cigarette smoking status | mild (AHI = 5–14), moderate (AHI = 15–30), and severe (AHI > 30) | Case 50.00±12.99 years  Control 45.73±13.91 years | - | - | No significant association was found between smoking and OSA | [7] |
| **Retrospective study** | 2020 | China | 1021 | Former smokers | AHI ≥5 with OSA-related symptoms; or AHI ≥15 with/without OSA-related symptoms | Case 46.7±12.3 years  Control 40.5±11.3 years | - | - | No significant association was found between previous smoking history and current OSA severity | [8] |
| **Cross-sectional study** | 2021 | Turkey | 384 | Smokers | AHI ≥ 5 with associated symptoms or AHI ≥ 15 with/without associated symptoms. | Case 44.99 ± 9.77 years  Control 50.83 ±11.29 years | - | - | Polysomnographic data did not yield statistically significant difference in non-smokers and smokers. OSA severity was not found correlated with smoking frequency. | [9] |
| **Retrospective cohort study** | 2021 | Greece | 3791 | Current smokers and former smokers | mild AHI 5-15/h, moderate AHI 15-30/h and severe AHI＞30/h. | 57.2 ± 13.6 years | - | - | Independent effect of smoking on OSA was not found, the number of cigarettes /day, Pack/Years, and FTND were higher in patients with more severe OSA with more prevalent CVD co-morbidities. | [10] |
| **Case-control study** | 2022 | China | 793 | Consume alcohol | polysomnographic study with more than 30 episodes of apnea–hypopnea during sleep 7 h per night or an AHI of ≥ 5 events/hour. | 57.4 ± 12.7  years | - | - | alcohol consumption was an independent risk factor of OSA and OSA with hypoxia | [11] |
| **Randomized controlled trial** | 2013 | Sweden | 73 | Participants with overweight, Prospective data of physical activity and sedentary time were collected through accelerometry | moderate/severe OSAS (apnea-hypopnea index ≥15) | 55±12 years | 10 days | 30 min of moderate  intensity activity 5 days a week | both physical activity and sedentary behaviors should be targeted, and fear of movement may be an important determinant for change in patients with OSA and overweight | [12] |
| **RCT** | 2021 | Brazil | 45 | Food intake evaluations | Full polysomnographic examinations | Case 41.8 ± 6.4 years  Control  39.6 ± 8.1 years | 1 month | low energy diet was  formulated to result in a 30% deficit in total energy expenditure  (between 500 and 800 kcal deficit per day) | One month of a low-energy diet resulted in significant improvements in OSA severity in obese men. Increased protein intake during a short period of low-energy diet had no further beneficial effects on OSA severity or biochemical parameters than a standard protein diet. | [13] |
| **Cross-sectional study** | 2011 | USA | 42 | Obese subjects and collection of the macronutrient content of dinners ordered from a standardized hospital menu the evening before the polysomnogram. | AHI was computed as the sum of relevant events divided by hours slept. | 10–16.9 years | - | - | More severe OSA appears to be associated with an increased preference for calorie-dense foods that are high in fat and carbohydrates in a manner that is independent of degree of obesity | [14] |
| **Cross-sectional study** | 2019 | Norway | 1200 | A proxy DSM-V insomnia diagnosis as well as an Insomnia Symptom Score (ISS, range 0-12) were calculated from three insomnia questions | AHI 5-14.9 (mild OSA), and AHI ≥ 15 (moderate-to-severe OSA). | Mean age Case 60.7 years  Control 55.4 years | - | - | In this population-based PSG study, no overall statistical association between OSA and insomnia prevalence was found. | [15] |
| **Cross-sectional study** | 2020 | USA | 727 | Sleep perception index (SPI) was defined by the ratio of subjective and objective values. | AHI 5.0-14.9/h (mild), 15.0-29.9/h (moderate), and ≥ 30.0/h (severe) | 46.1 ± 16.2 years | - | - | In a clinical referral cohort, SPI significantly decreases with increasing OSA severity, but is not modified by the presence of insomnia symptoms | [16] |
| **Cross-sectional study** | 2018 | Korea | 476 | Insomnia (Korean version of the Insomnia Severity Index score ≥ 15) | Polysomnographic test | 50.91 ±13.71 years | - | - | There is a high prevalence of comorbid insomnia with OSA (29.2%), consistent with previous findings in Western studies. | [17] |

CPAP, continuous positive airway pressure; AHI, apnea-hypopnea index; RDI, respiratory distress index; RCT, randomized controlled trial；

**Supplementary Table 8. Summary of the current studies examining the associations of serum parameters risk factors with OSA.**

| **Study design** | **Year** | **Country** | **Sample size** | **Status** | **Sleep apnea** | **Age** | **Follow-up duration** | **Intervention** | **Outcome** | **References** |
| --- | --- | --- | --- | --- | --- | --- | --- | --- | --- | --- |
| **Cross-sectional study** | 2022 | Taiwan | 326 | Serum CRP levels were measured | underwent an overnight polysomnography | 7.2 ± 3.0 years | - | - | Children with OSA had increased hs-CRP levels. Children with OSA and abnormal hs-CRP levels exhibited significantly reduced hs-CRP levels following adenotonsillectomy | [18] |
| **Retrospective cohort study** | 2022 | Serbia | 328 | Lipid abnormalities, inflammatory parameters | Mild AHI 5-15  Moderate AHI 15-30  Severe AHI≥30 | 54.0 ± 12.5 years | - | - | Inflammatory proteins CRP levels were higher in obese and hypertensive patients with OSA and CRP levels were higher in OSA with metabolic syndrome. | [19] |
| **Cross-sectional study** | 2022 | Italy | 110 | A polygraphic study and a blood draw for inflammatory markers were performed for each subject. | With a diagnosis of moderate or severe OSA | 45-80 years | - | - | A significant trend (p = 0.0001) for CRP levels from males with moderate OSA to females with severe OSA was shown. | [20] |
| **Cross-sectional study** | 2021 | Spain | 809 | Fasting blood samples were drawn to measure the lipid profile. | AHI: mild OSA (5-14.9/ h), moderate OSA (15-29.9/ h) or severe OSA (30/h) | Mild 58 ± 12 years; Moderate:60 ± 10 years; Severe 61 ± 11 years; Control 60 ± 12 years | - | - | The association between dyslipidemia and OSA is limited to severe patients, with high AHI and nocturnal hypoxemia. | [21] |
| **Cross-sectional study** | 2021 | Serbia | 104 | Severely obese (BMI) ≥ 35 kg/m^2^) men underwent sex hormone measurements | mild AHI 5-15/h, moderate AHI 15-30/h and severe AHI＞30/h. | 41.8 ± 11.9  years | - | - | OSA is associated with low total testosterone and free testosterone levels in severely obese men | [22] |
| **Cross-sectional study** | 2015 | Romania | 30 | Obese patients：total serum testosterone levels were determined  in blood samples, collected in the morning | AHI≥5 | Case  53 ± 6.6 years  Control  55 ± 5.9 years | - | - | OSA-related fatigue was strongly associated with serum testosterone, together with OSA severity | [23] |

AHI, apnea-hypopnea index; CRP, C-reactive protein; hs-CRP, high-sensitivity C-reactive protein

**Supplementary Table 9. Summary of the current studies examining the associations of metabolic comorbidities risk factors with OSA.**

| **Study design** | **Year** | **Country** | **Sample size** | **Status** | **Sleep apnea** | **Age** | **Follow-up duration** | **Intervention** | **Outcome** | **References** |
| --- | --- | --- | --- | --- | --- | --- | --- | --- | --- | --- |
| **Cross-sectional retrospective study** | 2017 | Canada | 30 | Underwent polysomnography and DEXA | AHI > 5 and obstructive AHI > 1 event/h | median age 14.1 years | - | - | NAF % ratio was associated with OSA severity in males and youth with BMI > 99th percentile | [24] |
| **Cross-sectional study** | 2020 | Lebanon | 25 | Diagnosed with Reinke's edema | Having two or more positive categories in the Berlin Questionnaire | Case  53.88 ±10.81 years  Control 48.32±16.83 years | - | - | Higher prevalence of snoring and obstructive sleep apnea in patients with Reinke's edema as evidenced by the Berlin Questionnaire. | [25] |
| **Cross-sectional study** | 2013 | Switzerland | 749 | Evaluation of body composition by DEXA | OSA (AHI ≥ 15) | 67.2 ± 0.8 years. | **-** | **-** | Central fat mass plays a role in the occurrence of severe OSAS in men older than 65 years of age. | [26] |
| **Cross-sectional study** | 2023 | Germany | 4420 | Quality of life was measured using the Short-Form 12 questionnaire  BMI | Mild AHI 5-15  Moderate AHI 15-30  Severe AHI≥30 | 53±14 years | - | - | Significant effects were found regarding age, sex, body mass index and the Short-Form 12 Mental Component Score | [27] |
| **RCT** | 2016 | USA | 359 | Non-diabetic participants with obesity  BMI 39.1 kg m(-2) | moderate (AHI 15-29.9 events h (-1)) or severe (AHI ⩾30 events h(-1)) | mean age 48.5 years | 32 weeks | 32 weeks to liraglutide 3.0 mg both as adjunct to diet (500 kcal day(-1) deficit) and exercise. | As an adjunct to diet and exercise, liraglutide 3.0 mg was generally well tolerated and produced significantly greater reductions than placebo in AHI, body weight, SBP and HbA1c in participants with obesity and moderate/severe OSA | [28] |
| **RCT** | 2017 | Australia | 60 | participants with obesity (BMI: >35 and <55) | AHI of ≥ 20 events/hour. | 47.7 (41.9, 52.8) | 2 years | low calorie diet with regular review or to laproscopic adjustable gastric banding | Following weight loss, a significant proportion (22%) of patients with obesity have normalization of the nonsupine AHI. | [29] |
| **RCT** | 2022 | Spain | 89 | BMI≥25 | moderate to severe OSA and receiving CPAP therapy (AHI ≥ 15). | Case 55.3±8.5 years  Control 52.6±7.1 years | 6 months | 8-week weight loss and lifestyle intervention involving nutritional behavior change, aerobic exercise, sleep hygiene, and alcohol and tobacco cessation | An interdisciplinary weight loss and lifestyle intervention with moderate to severe OSA and had overweight or obesity and were receiving CPAP therapy resulted in clinically meaningful and sustainable improvements in OSA severity and comorbidities as well as health-related quality of life. | [30] |
| **RCT** | 2021 | USA | 264 | BMI≥25 kg/m2 (or ≥27 kg/m2 if taking insulin), physician-verified type 2 diabetes, and HbA1C <11%. | mild (5 ≤ AHI <15 events/h)  moderate (15 ≤ AHI <30 events/h), severe (AHI ≥ 30 events/h) | 61.3±6.5 years | 10 years | Intensive Lifestyle Intervention for weight loss | Improvement in OSA severity over the 10-year period with ILI was related to change in body weight, baseline AHI, and intervention independent of weight change | [31] |
| **Prospective study** | 2016 | USA | 1453 | Non-diabetic participants of both | AHI <5.0 (normal), 5.0-14.9 (mild), 15.0-29.9 (moderate), and ≥30.0 events/h (severe) | average 62.5 years | 13 years | - | Severe obstructive sleep apnea was associated with greater risk of incident diabetes, independent of adiposity in a community-based sample | [32] |
| **Cross-sectional study** | 2021 | USA | 221 | Patients undergoing a 3-h oral glucose tolerance test (OGTT) and a two-step hyperglycemic clamp | OSA was graded as absent, mild, moderate, or severe if AHI was <5, 5–14, 15–29, or ≥30 events per hour | 54.5 ± 8.7 years | - | - | OSA severity and sleep duration were not associated with measures of insulin sensitivity or β-cell responses | [33] |
| **Cross-sectional study** | 2011 | USA | 200 | Patients with a diagnosis of hypertension provided subjective and clinical data | OSA defining high risk as a total ARES score ≥6 | 63 ± 13 years | - | - | Patients with resistant hypertension were nearly 2.5 times more likely to be at high OSA risk, relative to those with hypertension | [34] |
| **Cross-sectional study** | 2006 | Spain | 49 | Patients treated in hospital with suspected difficult-to-control hypertension | mild AHI 10-29 severe AHI>30 | 68.1 ± 9.1 years | - | - | Prevalence of SAHS was very high in patients with difficult-to-control hypertension | [35] |
| **Case-control study** | 2002 | Sweden | 102 | Patient was diagnosed with hypertension | mean (SD) AHI was 9.0 (10.8). | Case 60.6±10.0 years  Control 60.7±9.6 years | - | - | SDB is more prevalent in men with hypertension than in controls | [36] |
| **Cross-sectional study** | 2010 | Spain | 62 | 24-h blood pressure (BP) in patients with resistant hypertension, defined as clinic BP values ≥ 140/90 | OSA was defined as an AHI ≥ 5 | 59 ± 10  years | - | - | A high prevalence of severe OSA in patients with resistant hypertension | [37] |
| **Cross-sectional study** | 2011 | Saudi Arabia. | 271 | Serum thyroid-stimulating hormone and free-thyroxine (FT4) levels were measured | mild AHI 5-15/h, moderate AHI 15-30/h and severe AHI＞30/h. | Case 53.3 ± 14.4 years  Control 47.8±14 years | - | - | In the OSA patients, the prevalence of newly diagnosed clinical hypothyroidism was low; however, subclinical hypothyroidism was common among patients with OSA | [38] |

ARES, Apnea Risk Evaluation System; BMI, body mass index; CPAP, continuous positive airway pressure; DEXA, dual-energy X-ray absorbsiometry; AHI, apnea-hypopnea index; NAF, neck-to-abdominal fat percentage; RCT, randomized controlled trial；SBP, systolic blood pressure; HbA1c, glycated hemoglobin；SAHS, sleep apnea-hypopnea syndrome; SDB, sleep disordered breathing.
