# Supplementary material for: Association of modifiable risk factors with obstructive sleep apnea: a Mendelian randomization study
Source: Aging (Albany NY). 2023 Dec 11;15(23):14039–65. doi: 10.18632/aging.205288 (PMC10756101; doi:10.18632/aging.205288)
Supplement: Supplementary References [file aging-15-205288-s004.pdf]

SUPPLEMENTARY REFERENCES

1. Alchanatis M, Zias N, Deligiorgis N, Amfilochiou A, Dionellis G, Orphanidou D. Sleep apnea-related cognitive deficits and intelligence: an implication of cognitive reserve theory. J Sleep Res. 2005; 14:69–75. https://doi.org/10.1111/j.1365-2869.2004.00436.x PMID:15743336

2. Hlaing EE, Dollinger SMC, Brown TM. The role of education in cognitive functions among middle-age and older patients with untreated obstructive sleep apnea. Sleep Sci. 2021; 14:319–29. https://doi.org/10.5935/1984-0063.20200099 PMID:35087628

3. Abulhamail A, AlShebli A, Merdad L, Wali S, Jastaniah W, Abaalkhail B. Prevalence of and risk factors for obstructive sleep apnea in children with sickle cell: a multicentric cross sectional study. Ann Hematol. 2022; 101:43–57. https://doi.org/10.1007/s00277-021-04646-1 PMID:34505943

4. Gliklich RE, Taghizadeh F, Winkelman JW. Health status in patients with disturbed sleep and obstructive sleep apnea. Otolaryngol Head Neck Surg. 2000; 122:542–6. https://doi.org/10.1067/mhn.2000.102579 PMID:10740175

5. Duan X, Zheng M, He S, Lao L, Huang J, Zhao W, Lao XQ, Deng H, Liu X. Association between physical activity and risk of obstructive sleep apnea. Sleep Breath. 2021; 25:1925–34. https://doi.org/10.1007/s11325-021-02318-y PMID:33585989

6. Kline CE, Crowley EP, Ewing GB, Burch JB, Blair SN, Durstine JL, Davis JM, Youngstedt SD. The effect of exercise training on obstructive sleep apnea and sleep quality: a randomized controlled trial. Sleep. 2011; 34:1631–40. https://doi.org/10.5665/sleep.1422 PMID:22131599

7. Hsu WY, Chiu NY, Chang CC, Chang TG, Lane HY. The association between cigarette smoking and obstructive sleep apnea. Tob Induc Dis. 2019; 17:27. https://doi.org/10.18332/tid/105893 PMID:31582938

8. Shao C, Qi H, Fang Q, Tu J, Li Q, Wang L. Smoking history and its relationship with comorbidities in patients with obstructive sleep apnea. Tob Induc Dis. 2020; 18:56. https://doi.org/10.18332/tid/123429 PMID:32641922

9. Esen AD, Akpinar M. Relevance of obstructive sleep apnea and smoking: Obstructive sleep apnea and smoking. Fam Pract. 2021; 38:181–6. https://doi.org/10.1093/fampra/cmaa112 PMID:33128376

10. Ioannidou D, Kalamaras G, Kotoulas SC, Pataka A. Smoking and Obstructive Sleep Apnea: Is There An Association between These Cardiometabolic Risk Factors?-Gender Analysis. Medicina (Kaunas). 2021; 57:1137. https://doi.org/10.3390/medicina57111137 PMID:34833356

11. Yang S, Guo X, Liu W, Li Y, Liu Y. Alcohol as an independent risk factor for obstructive sleep apnea. Ir J Med Sci. 2022; 191:1325–30. https://doi.org/10.1007/s11845-021-02671-7 PMID:34110582

12. Igelström H, Emtner M, Lindberg E, Asenlöf P. Physical activity and sedentary time in persons with obstructive sleep apnea and overweight enrolled in a randomized controlled trial for enhanced physical activity and healthy eating. Sleep Breath. 2013; 17:1257–66. https://doi.org/10.1007/s11325-013-0831-6 PMID:23536259

13. de Melo CM, Dos Santos Quaresma MVL, Del Re MP, Ribeiro SML, Moreira Antunes HK, Togeiro SM, Tufik S, de Mello MT. One-month of a low-energy diet, with no additional effect of high-protein, reduces Obstructive Sleep Apnea severity and improve metabolic parameters in obese males. Clin Nutr ESPEN. 2021; 42:82–9. https://doi.org/10.1016/j.clnesp.2020.12.028 PMID:33745625

14. Beebe DW, Miller N, Kirk S, Daniels SR, Amin R. The association between obstructive sleep apnea and dietary choices among obese individuals during middle to late childhood. Sleep Med. 2011; 12:797–9. https://doi.org/10.1016/j.sleep.2010.12.020 PMID:21871836

15. Uhlig BL, Hagen K, Engstrøm M, Stjern M, Gravdahl GB, Sand T. The relationship between obstructive sleep apnea and insomnia: a population-based cross-sectional polysomnographic study. Sleep Med. 2019; 54:126–33. https://doi.org/10.1016/j.sleep.2018.10.026 PMID:30554056

16. Duarte RLM, Mendes BA, Oliveira-E-Sá TS, Magalhães-da-Silveira FJ, Gozal D. Perception of sleep duration in adult patients with suspected obstructive sleep apnea. PLoS One. 2020; 15:e0238083.

https://doi.org/10.1371/journal.pone.0238083
PMID:32853299

17. Cho YW, Kim KT, Moon HJ, Korostyshevskiy VR, Motamedi GK, Yang KI. Comorbid Insomnia With Obstructive Sleep Apnea: Clinical Characteristics and Risk Factors. J Clin Sleep Med. 2018; 14:409–17.
https://doi.org/10.5664/jcsm.6988
PMID:29458695

18. Kang KT, Weng WC, Lee PL, Hsu WC. C-reactive protein in children with obstructive sleep apnea and effects of adenotonsillectomy. Auris Nasus Larynx. 2022; 49:92–9.
https://doi.org/10.1016/j.anl.2021.06.001
PMID:34272056

19. Popadic V, Brajkovic M, Klasnja S, Milic N, Rajovic N, Lisulov DP, Divac A, Ivankovic T, Manojlovic A, Nikolic N, Memon L, Brankovic M, Popovic M, et al. Correlation of Dyslipidemia and Inflammation With Obstructive Sleep Apnea Severity. Front Pharmacol. 2022; 13:897279.
https://doi.org/10.3389/fphar.2022.897279
PMID:35694268

20. Rocchi C, Valentina C, Totaro V, Broggi S, Lattanzi S, Viticchi G, Falsetti L, Silvestrini M, Buratti L. Inflammation markers in moderate and severe obstructive sleep apnea: the influence of sex. Sleep Breath. 2022; 26:1703–9.
https://doi.org/10.1007/s11325-021-02537-3
PMID:34988848

21. Martínez-Cerón E, Casitas R, Galera R, Sánchez-Sánchez B, Zamarrón E, Garcia-Sanchez A, Jaureguizar A, Cubillos-Zapata C, Garcia-Rio F. Contribution of sleep characteristics to the association between obstructive sleep apnea and dyslipidemia. Sleep Med. 2021; 84:63–72.
https://doi.org/10.1016/j.sleep.2021.05.012
PMID:34111805

22. Tančić-Gajić M, Vukčević M, Ivović M, Marina LV, Arizanović Z, Soldatović I, Stojanović M, Đogo A, Kendereški A, Vujović S. Obstructive Sleep Apnea Is Associated With Low Testosterone Levels in Severely Obese Men. Front Endocrinol (Lausanne). 2021; 12:622496.
https://doi.org/10.3389/fendo.2021.622496
PMID:34381420

23. Bercea RM, Mihaescu T, Cojocaru C, Bjorvatn B. Fatigue and serum testosterone in obstructive sleep apnea patients. Clin Respir J. 2015; 9:342–9.
https://doi.org/10.1111/crj.12150
PMID:24725752

24. Glicksman A, Hadjiyannakis S, Barrowman N, Walker S, Hoey L, Katz SL. Body Fat Distribution Ratios and Obstructive Sleep Apnea Severity in Youth With Obesity. J Clin Sleep Med. 2017; 13:545–50.
https://doi.org/10.5664/jcsm.6538
PMID:28095969

25. Hamdan AL, Khalifee E, Abi Akl PR, Ghanem A, El Hage A. Pathogenic Role of Reinke's Edema in Snoring and Obstructive Sleep Apnea. J Voice. 2020; 34:456–9.
https://doi.org/10.1016/j.jvoice.2018.12.001
PMID:30563731

26. Degache F, Sforza E, Dauphinot V, Celle S, Garcin A, Collet P, Pichot V, Barthélémy JC, Roche F, and PROOF Study Group. Relation of central fat mass to obstructive sleep apnea in the elderly. Sleep. 2013; 36:501–7.
https://doi.org/10.5665/sleep.2532
PMID:23564997

27. Vogler K, Daboul A, Obst A, Fietze I, Ewert R, Biffar R, Krüger M. Quality of life in patients with obstructive sleep apnea: Results from the study of health in Pomerania. J Sleep Res. 2023; 32:e13702.
https://doi.org/10.1111/jsr.13702
PMID:36053870

28. Blackman A, Foster GD, Zammit G, Rosenberg R, Aronne L, Wadden T, Claudius B, Jensen CB, Mignot E. Effect of liraglutide 3.0 mg in individuals with obesity and moderate or severe obstructive sleep apnea: the SCALE Sleep Apnea randomized clinical trial. Int J Obes (Lond). 2016; 40:1310–9.
https://doi.org/10.1038/ijo.2016.52
PMID:27005405

29. Joosten SA, Khoo JK, Edwards BA, Landry SA, Naughton MT, Dixon JB, Hamilton GS. Improvement in Obstructive Sleep Apnea With Weight Loss is Dependent on Body Position During Sleep. Sleep. 2017; 40.
https://doi.org/10.1093/sleep/zsx047
PMID:28444355

30. Carneiro-Barrera A, Amaro-Gahete FJ, Guillén-Riquelme A, Jurado-Fasoli L, Sáez-Roca G, Martín-Carrasco C, Buela-Casal G, Ruiz JR. Effect of an Interdisciplinary Weight Loss and Lifestyle Intervention on Obstructive Sleep Apnea Severity: The INTERAPNEA Randomized Clinical Trial. JAMA Netw Open. 2022; 5:e228212.
https://doi.org/10.1001/jamanetworkopen.2022.8212
PMID:35452108

31. Kuna ST, Reboussin DM, Strotmeyer ES, Millman RP, Zammit G, Walkup MP, Wadden TA, Wing RR, Pi-Sunyer FX, Spira AP, Foster GD, and Sleep AHEAD Research Subgroup of the Look AHEAD Research Group. Effects of Weight Loss on Obstructive Sleep

Apnea Severity. Ten-Year Results of the Sleep AHEAD Study. Am J Respir Crit Care Med. 2021; 203:221–9. https://doi.org/10.1164/rccm.201912-2511OC PMID:32721163

32. Nagayoshi M, Punjabi NM, Selvin E, Pankow JS, Shahar E, Iso H, Folsom AR, Lutsey PL. Obstructive sleep apnea and incident type 2 diabetes. Sleep Med. 2016; 25:156–61. https://doi.org/10.1016/j.sleep.2016.05.009 PMID:27810258

33. Mokhlesi B, Tjaden AH, Temple KA, Edelstein SL, Sam S, Nadeau KJ, Hannon TS, Manchanda S, Mather KJ, Kahn SE, Ehrmann DA, Van Cauter E, and RISE Consortium. Obstructive Sleep Apnea, Glucose Tolerance, and β-Cell Function in Adults With Prediabetes or Untreated Type 2 Diabetes in the Restoring Insulin Secretion (RISE) Study. Diabetes Care. 2021; 44:993–1001. https://doi.org/10.2337/dc20-2127 PMID:33547205

34. Demede M, Pandey A, Zizi F, Bachmann R, Donat M, McFarlane SI, Jean-Louis G, Ogedegbe G. Resistant hypertension and obstructive sleep apnea in the primary-care setting. Int J Hypertens. 2011; 2011:340929. https://doi.org/10.4061/2011/340929 PMID:21755035

35. Martínez-García MA, Gómez-Aldaraví R, Gil-Martínez T, Soler-Cataluña JJ, Bernácer-Alpera B, Román-Sánchez P. Trastornos respiratorios durante el sueño en pacientes con hipertensión arterial de difícil control [Sleep-disordered breathing in patients with difficult-to-control hypertension]. Arch Bronconeumol. 2006; 42:14–20. https://doi.org/10.1016/s1579-2129(06)60108-0 PMID:16426518

36. Sjöström C, Lindberg E, Elmasry A, Hägg A, Svärdsudd K, Janson C. Prevalence of sleep apnoea and snoring in hypertensive men: a population based study. Thorax. 2002; 57:602–7. https://doi.org/10.1136/thorax.57.7.602 PMID:12096203

37. Lloberes P, Lozano L, Sampol G, Romero O, Jurado MJ, Ríos J, Untoria MD, Tovar JL. Obstructive sleep apnoea and 24-h blood pressure in patients with resistant hypertension. J Sleep Res. 2010; 19:597–602. https://doi.org/10.1111/j.1365-2869.2010.00839.x PMID:20545837

38. Bahammam SA, Sharif MM, Jammah AA, Bahammam AS. Prevalence of thyroid disease in patients with obstructive sleep apnea. Respir Med. 2011; 105:1755–60. https://doi.org/10.1016/j.rmed.2011.07.007 PMID:21820299
